# Nutrient Intakes in Vegans, Lacto-Ovo-Vegetarians, Orthodox Fasters, and Omnivores in Russia: A Cross-Sectional Study

**DOI:** 10.3390/foods14061062

**Published:** 2025-03-20

**Authors:** Alexey Vladimirovich Galchenko, Gianluca Rizzo, Luciana Baroni

**Affiliations:** 1 Scientific Society for Vegetarian Nutrition—SSNV, Mestre, 30171 Venice, Italy; luciana.baroni@scienzavegetariana.it; 2 Earth Philosophical Society “Melodia Vitae”, Toronto, ON M9A4X9, Canada; 3 Independent Researcher, 98121 Messina, Italy

**Keywords:** vitamin, macro element, trace element, plant-based, Orthodox Great Lent

## Abstract

In Eastern Europe, the number of vegetarians is growing, and the number of people adhering to Christian Lents is traditionally high. However, data on the nutritional value of plant-based diets in this part of the world are limited. The aim of this study was to compare the nutritional intakes of three groups with different plant-based patterns with that of omnivores in Russia, Moscow region. The nutrient intakes of 46 vegans, 49 lacto-ovo-vegetarians, 42 people who adhered to Orthodox Great Lent, and 48 omnivores were assessed. The food frequency questionnaire method was used for data collection and analysis. The differences in absolute and calorie adjusted nutrient intakes between the groups were analysed. Additionally, a pairwise comparison of the general plant-based group (combined of the vegan, lacto-ovo-vegetarian, and Great Lent samples) and the omnivorous groups was conducted. Vegan diet was the most favourable in micronutrient composition. The intake of many micronutrients increased when switching to a more plant-based diet from a more animal-based one. The opposite association was observed only for selenium and vitamins D and B_12_. Fasting people consumed more iodine and n-3 polyunsaturated fatty acids; however, after the calorie content was standardized, the omnivores caught up with them. The omnivores had the largest list of dietary inadequacies: they significantly more often than all other groups had inadequate intake of cholesterol (excessive), fibre, potassium, magnesium, iron, and vitamins B_1_, B_6_, B_9_, and E (insufficient). Inadequate intake of polyunsaturated fatty acids, calcium, iodine, chromium, molybdenum, and zinc; or vitamins B_2_, PP, H, B_12_, and D was observed rather often in all the studied groups. Although, the vegan diet was richer in most micronutrients, plant products often contain substances that reduce the bioavailability of various nutrients, which can partially affect their status in the body, and, thus, may increase the need in them in vegetarians and fasters.

## 1. Introduction

The number of people following a plant-based diet is constantly growing worldwide [1,2]. Plant-based diets encompass different types, depending on the products consumed, and the most common are vegetarian diets (i.e., vegan, VN, and lacto-ovo, LOV) [3]. All vegetarian diets avoid the intake of meat, poultry, fish and seafood, as well as slaughterhouse byproducts (gelatine, animal fat, lard or rennet) [4]. At the same time, the LOV diet retains eggs and dairy products [5]. VNs avoid all animal products, including honey. However, all vegetarians consume a large variety of plant-based foods [4,6,7].

VNs and LOVs stick to their diet for different reasons. These include health care and disease prevention [8,9], cultural beliefs, ethical reasons (concern for animal rights), and environmental care [8,10,11]. Many of them also limit their diet for religious reasons [10,12]. Recently, it has been proposed that the extensive exclusion of foods of animal origin could offer a sustainable approach to addressing the growing environmental impact of human activities, ensuring sustainability for the environment and the health of populations worldwide [13].

Other types of plant-based diets are those in which people adhere to certain periodic food restrictions on the basis of religious considerations—a Lent [10,12]. There is a great variety of Lents depending on the specific religion [14]. In Orthodoxy, there are the Great, Assumption, Apostles’ and Christmas Lents [15]. The Great Lent lasts 48 days and is based on the avoidance of slaughter food (meat, poultry, and fish) and other products derived from animals (dairy products, eggs), and alcohol; the diet consists mainly of plant foods. Plant oils and wine are allowed only on Saturdays and Sundays except Saturday before Easter [15]. Despite that oil consumption is strictly allowed only two days per week, the majority of the population avoids this role, and consumes plant oils without restrictions, considering them simple products, unlike they were centuries ago [16].

Fish is allowed on Annunciation Day, Lazarev Saturday and Palm Sunday [15,17]. At the same time, the question of whether sea invertebrates are eaten remains unclear: some believe that they can be eaten without restriction [18], whereas others are sure that sea invertebrates can be allowed on the same days as fish is allowed [16] or on Saturdays and Sundays as oils [16,19]. In general, given that the purpose of the Lent is spiritual education, sea invertebrates may be allowed if they are a familiar food for a person and not a delicacy; otherwise, they should be discarded [16,19,20]. A person is often free to adjust the features of a fasting diet to his own needs, lifestyle and health [16,19].

Many believers also adhere to a Lent on Wednesdays and Fridays during the whole year [15]. Fasting people (FSs) mainly eat vegetables, fruits, grains, pulses, seeds, nuts, and honey. Thus, the diet during the Orthodox Lent is a plant-based diet intermediate between veganism and pescetarianism [14].

The different sets of foods in different types of diets also determine the different nutrient composition of these diets. The complete exclusion of certain foods without their adequate replacement can lead to a sharp decrease in the intake of various micronutrients. Thus, concerns remain about potential nutritional deficiencies in plant-based diets, particularly in n3-PUFAs [21,22,23], calcium [24], iron [25], zinc [26], selenium [27], vitamin B_12_ [28], and even vitamin D [29]. Among LOVs and VNs, less favourable levels of certain blood markers, such as elevated homocysteine and methylmalonic acid, along with decreased levels of serum vitamin B_12_ and holotranscobalamin, are observed when compared to omnivores (OMN) [30]. Without adequate supplementation, reduced intake of these micronutrients could negate the benefits of a plant-based diet, with possible repercussions on bone health [31,32] or haematopoesis [33]. The risks of bone loss are also present in individuals practicing intermittent fasting, including fasting for religious purposes [34]. Makedou et al. [35] found a decrease in almost all general blood test parameters in FSs right before the Easter compared to the levels before the Great Lent. An Ethiopian study [36] in lactating women showed a growth retardation in their children in the Lent periods. Several Russian studies reported dyspepsia, skin disorders, and frequent colds [37], as well as low vitamins B_1_, B_2_, and B_6_ serum concentrations [38] in FSs during the Lent.

At the same time, adopting a plant-based diet may increase the dietary content of certain compounds, which are deficient in other diets or, vice versa, reduce the intake of commonly excessive nutrients. Series of Sarri et al. studies [39,40,41] showed a significant improvement of the lipid profile in all three large Orthodox Lents (Christmas, Great, and Assumption). The same was observed after the Danilov Lent (21 days) [42]. A 2018 study [35] found an increase of total antioxidant capacity after the FS period. Several reviews conclude that the Christian Lent has in general a good effect on human health, mainly normalizing macronutrients consumption [14,43]. Chliaoutakis et al. [44] noted that those who adhere to religious Lents usually have a healthier lifestyle. In addition, the authors believe that religious people have a more positive psycho-emotional condition, which may influence also the somatic health through psychosomatic pathways.

LOV and VN diets have proven efficacy in reducing mortality from ischemic stroke and ischemic heart disease, as well as in lowering the risks of cerebrovascular and cardiovascular diseases incidence [45]. Additionally, plant-based diets have demonstrated beneficial effects on blood lipid profile [46], body weight management [47], insulin sensitivity [47], gut microbiota composition [48], and periodontitis [49,50,51,52]. Moreover, substituting cow’s milk with soy milk has shown advantages for inflammation, blood lipid levels, and blood pressure without adverse cardiometabolic effects [53]. Another emerging benefit of a vegetarian diet is its potential positive impact on the progression of chronic kidney disease [54,55]. And finally, a 2024 meta-analysis did not show elevated risks for the discussed above nutritional deficiencies in VNs and LOVs, whether they adequately supplement their diet and consume fortified foods [56].

As a kind of a plant-based diet, the FS diet has many similarities with other, much better studied types of plant-based diets, first of all, VN and LOV. At the same time, they are usually studied separately and we could find not a single study where the FS diet was simultaneously analysed with any of the vegetarian ones. Moreover, despite the extensive availability of nutritional and health data on plant-based diets, current information remains highly heterogeneous. Another concern is a severe lack of knowledge on the nutritional status of both VNs, LOVs, and FSs in the Eastern Europe. While the majority of studies in vegetarians were conducted in the EU and the UK states, the USA, or Eastern and South-Eastern Asia, most results on the FS effects came from Greece.

Religious, cultural, and economical features of different parts of the world, as well as their geographical characteristics, primarily, biogeochemical conditions, necessitate the spread of comprehensive research into the nutrition and health conditions of plant-based diets followers in less investigated in this regard regions. Russia is one of those regions that extremely lacks data both on vegetarians and FSs.

We aimed to cross-sectionally compare the nutrient contents of two vegetarian diets—VN and LOV—and a type of a FS diet—the Orthodox Great Lent—with the OMN diet in Moscow adult citizens. We expect our results to be useful for dietetic and medical practitioners in Eastern Europe, providing them a deeper insight into the nutritional daily intakes among VNs, LOVs, and FSs, as well as the adequacy of these plant-based diets relatively to the OMN one.

## 2. Materials and Methods

### 2.1. Study Population

A total of 185 adult participants were enrolled in the study: 46 VNs, 49 LOVs, and 48 OMNs were examined at the Clinic of the Federal Research Centre of Nutrition, Biotechnology, and Food Safety, Moscow. VNs and LOVs were invited to participate in the study through social media and vegetarian fests in person. Those who did not consume any animal products for at least one year before the enrolment were categorized as VNs, and those who did not consume any slaughter food (meat, poultry, fish, invertebrates and slaughterhouse byproducts) were categorized as LOVs. Forty-two FSs were recruited at St. Alexis’s Central Clinical Hospital, Moscow, among the staff of the Hospital, during the last week of the Orthodox Great Lent in April 2018 and were examined there. OMNs were invited to participate in the study last to match the rest of the groups in terms of age and gender composition, as well as anthropometric parameters. Social media were used in this case as well. LOVs and VNs were invited to the study from February 2018 to July 2019, and OMNs were invited from May 2019 to December 2019. We did not include to the samples subjects affected by serious somatic, neurological, or psychiatric pathologies.

The sample size was calculated intensively within the black box model, considering the criteria power of 0.9, confidence interval 0.95, variety < 0.4, and φ < 0.1. For the extensive analysis the macronutrient bias from the Sobiecki et al. [57] data was used. The calculation was carried out with the WinPepi program (Brixton Health, UK). The minimal effective size was calculated as 137 subjects (185 subjects were recruited).

### 2.2. Ethical Statement

Voluntary written informed consent was obtained in advance from all participants. The study was approved by the Ethics Committee of the Federal Research Centre of Nutrition, Biotechnology and Food Safety (protocol No. 6 from 22.12.2017, Moscow). This study was conducted in accordance with the Helsinki Declaration of the World Medical Association (1964) and its subsequent amendments.

### 2.3. Dietary Assessment

Nutrition assessment of the subjects was performed via the Nutrilogic program (Nutrilogic LLC, Moscow, Russia). The Nutrilogic software (2025) is based on the food composition database “The Chemical Composition and Caloric Content of Russian Food” [58], which was complied in accordance to the EUROCOD guidelines [59]. The authors of the database used methods recommended by the «Guide to Food Quality and Safety Analysis Methods» [60] and the «Guide to Food Supplements Quality and Safety Analysis Methods» [61]. The efficiency of the Nutrilogic service for the analysis of human nutrition and the chemical composition of diets and its validity were confirmed in 2018 at the Moscow State University of Food Production [62].

The Food Frequency Questionnaire (FFQ) method was chosen since the nutrient composition of the diet (except for FSs) was estimated for one year. FS nutrient intake was evaluated only for the period of the Great Lent (48 days). This is why there were no seasonal products (fruits or vegetables) in these subjects’ diets. Products available during the Lent period (March–April) were taken into account in the analysis. One interview lasting 40–70 min was conducted with each of the subjects. Information about nutrition was collected by a dietician (A.V.G.) at the Clinic of the Federal Research Centre of Nutrition, Biotechnology, and Food Safety and St. Alexis’ Central Clinical Hospital. The validity of the FFQ method was shown in previous studies [63,64,65].

The interview was fully structured. A few days before the interview, each of the subjects was asked to recall the features of their diet and the frequency of consumption of the main dishes and products. During the analysis itself, the interviewer offered to evaluate the consumption of each product separately: whether the subject consumed this product during the year, how often and in which quantity. The following intervals of food consumption frequency were offered: “every day”, “2–4 times a week”, “once a week”, “twice a month”, “once a month”, and “2–4 times a year”.

The amount of food consumed was estimated in grams via photographic measures. For this purpose, colour images of portions of known weights of various products and dishes included in the Nutrilogic program package were used. The life-size portions of different weights were presented in photographs on the monitor screen, and the subject determined how much his/her average portion was commensurate with any of the shown ones.

Fortified food because of its scarce diffusion in Russia and the absence of mandatory food fortification is not included in the Nutrilogic software (2025) database. Therefore, the intake of a fortified product, if any, was calculated as not fortified. As the goal of the study was to evaluate the diets themselves, we did not consider supplement use in our calculations.

### 2.4. Statistical Analysis

The calculations and statistical processing of the results were performed via SPSS program v. 23.0.0.0 (IBM, Chicago, IL, USA). Absolute nutrient consumption values were calculated for each group. The parameter “n-6:n-3 ratio” was additionally calculated as the consumption ratio. Finally, absolute values for the lower (1st—25th percentile) and upper (75th—100th percentile) quartiles were calculated. The Shapiro–Wilk test was used to evaluate the distribution of the values.

The results obtained were compared with the reference intake values of nutrient consumption established in Russia [66,67]. Reference values for the indicator “fibre” are significantly different in the USA [68] and in the Russian Federation [66]; thus, we conducted an additional analysis using the US recommendations for fibre consumption. The percentage of people with inadequate intake of nutrients in each group was calculated. The percentages of subjects with deviations between groups were compared via Fisher’s exact test, the χ^2^test, or Yates’s χ^2^test, depending on the frequency table.

To compare the plant-based and omnivorous dietary patterns as a whole, VNs, LOVs, and FSs were combined into one group, and then, the group was compared with OMNs.

To exclude the effect of total food intake, nutrient intakes were additionally estimated in relation to the total calorie value of the subjects’ diets. The distribution of the energy values of the diets among all the subjects was not Gaussian. Consequently, the median value of this indicator (1948 kcal) was determined. The diets of all the subjects were subsequently recalculated to the median total calorie value. Gender, age, and body mass index (BMI) adjustments were applied to diminish the impact of these covariables.

Descriptive data for quantitative parameters are presented as medians and interquartile ranges (25th; 75th percentiles). In the case of a normal distribution of an indicator, all 4 groups were compared via ANOVA, whereas paired comparisons of independent variables were made via Student’s t test for independent groups. In the case of an abnormal distribution, the Kruskal–Wallis H test and Mann–Whitney U test were used for independent variables. The Holm-Bonferroni correction was applied when multiple comparisons were present. One way Spearman correlation test was used then to find linear associations of the diet group with nutrient intakes. To determine the influence of independent input variables on nutrient intakes and calorie adjusted nutrient intakes, a two-factor (diet + age) and a three-factor (diet + gender + BMI) rank analyses of variance of the 4 × 2 and 4 × 2 × 2 types, respectively, were carried out. Adjustment for gender, age, and BMI analysis of the 2^4^ type with two levels on the diet variable (plant-based and OMN) was performed within Yates’ analysis.

## 3. Results

The anthropometric parameters of the samples as well as their age and gender distribution are presented in Supplementary Table S1.

The effect of gender (I and Co) and BMI (protein, fat, saturated fatty acids (SFAs), n-3 polyunsaturated fatty acids (PUFAs), cholesterol, fibre, P, Se, Zn, vitamins B_12_ and D) on absolute nutrient intakes was expected, however, these factors had a significantly smaller impact on calorie adjusted nutrient intakes. The results of multifactor analysis of variance are presented in Supplementary Tables S2 and S3. n-6:n-3 PUFAs ratio, and calorie adjusted n-3 PUFAs, cholesterol, and vitamins B_12_ and D were associated with BMI, but only a weak linear dependence (r < 0.3) was observed (Supplementary Table S4).

The results of the evaluation of caloric intake, different nutrient intakes and n-6:n-3 PUFAs ratios depending on the type of diet are shown in Table 1. The caloric intakes of FSs and VNs were higher than those of OMNs, and the LOVs were in an intermediate position.

Both FSs and OMNs consumed more protein than VNs and LOVs did. The OMN diet was the richest in SFAs, while monounsaturated fatty acids (MUFAs) intake was greater in the VN diet, and total PUFAs intake was greater in the LOV and VN diets. The n-6:n-3 PUFAs ratio had better values for OMNs and FSs than for VNs and LOVs. VNs and FSs consumed the most carbohydrates, mono- and disaccharides (MDS) and fibre.

The VN diet was the source of the highest amounts of K and Mg, followed by the FS and LOV diets. LOVs consumed more Ca than FSs and OMNs did.

VNs consumed the most Fe, Co, Cu, and OMNs consumed the least amount of these nutrients. Cr and Mo were also consumed the most in VNs and the least in FSs and OMNs. Compared with the other groups, the LOV diet had the smallest amount of I and Zn. The major consumers of Se were OMNs and FSs.

VNs and FSs consumed the greatest amounts of vitamins B_1_ and PP. VNs consumed more vitamins B_5_, B_6_, H, and C than the other groups did. VNs and FSs were also major consumers of vitamin B_9_. Notably, LOVs were closer to OMNs by folate intake than to VNs. In plant-based diets, there was almost no vitamin B_12_, but even OMNs consumed insufficient amount of it.

VNs consumed the largest amounts of vitamin A, expressed as a retinol equivalent (RE), while FSs had the lowest level of its intake. All groups had relevant vitamin D inadequacy. OMNs consumed the smallest amount of vitamin E (tocopherol equivalent—TE).

Table 2 shows the percentage of nutrient intake inadequacy in each group. All groups had a very high risk of inadequate consumption of n-3 PUFAs, especially VNs and LOVs. Also, all subjects had a fairly high inadequacy of n-6 PUFAs, but the highest one was among OMNs. More than a quarter of FSs and OMNs had an offset of the n-6:n-3 ratio to n-3 PUFAs, whereas VNs and LOVs, in contrast, were more likely to be shifted to n-6 PUFAs.

The prevalence of Ca intake inadequacy was comparable between the groups, in more than two-thirds of the subjects and 90% among OMNs. OMNs tended to have the highest percentage of Mg intake inadequacy, whereas VNs—the lowest. K intake inadequacy was the most spread among OMNs.

Inadequate intake of Fe was observed in more than half of OMNs, as well as insufficient Cu intake in 1/3 of them. No more than 7% of VNs had insufficient intake of Fe, Cu, and Co. VNs and LOVs had a high rate of inadequacy for Se intake. The prevalence of I, Mo, Cr, and Zn inadequacies was high among all the groups. Co intake inadequacy was moderate, whereas Mn inadequacy was low. Interestingly, 1 person among FSs consumed more than 600 mg of I (upper limit).

The risks of inadequate intake of vitamins B_2_, PP, and H were relatively high in all groups. The results for biotin inadequacy were particularly remarkable: 91% of VNs, 98% of FSs, and 100% of LOVs and OMNs consumed inadequate amounts of vitamin B_7_. Adherents of plant diets also consumed critically low levels of vitamin B_12_. Notably, among OMNs, the risk of inadequate intake was also high (69%). OMNs had the highest prevalence of inadequate intake of vitamins B_1_, B_5_, B_6_, and B_9_, and VNs had the lowest prevalence. LOVs and FSs also had a high frequency of inadequacy for vitamins B_5_ and B_6_. No VN consumed vitamin C below the recommended intake, unlike subjects from other groups.

The RE inadequacy in the diet was quite high among all groups, except VNs. The risk of vitamin D inadequacy was absolute regardless of the type of diet. One-third of all LOVs and FSs and 2/3 of OMNs consumed inadequate amounts of vitamin E.

When the groups were divided by gender, the patterns of the total sample were maintained, whereas there were greater risks of inadequate consumption of fibre in men (except for VNs) and Fe in women, which was due to significant gender differences in the reference values of physiological needs for these nutrients.

When the excess consumption of nutrients was assessed, it was found that OMNs had the lowest prevalence of excess MDS in the diet but the highest rate of cholesterol excess.

Supplementary Table S5 displays nutrient consumption in the lower quartiles. Compared with the total sample, there were no differences in PUFAs consumption between groups in the lower quartile. The n-6:n-3 ratio decreased in all groups towards n-3 PUFAs. The OMN diet in the lower quartile was the richest in I, and the consumption of Cu was equal to the LOV diet. Zn consumption decreased significantly in LOVs relative to those of the other groups and in contrast to the total sample group comparison. The amount of vitamin A in the VN diet was reduced, and they came closer to the other groups. For the rest, the patterns in the total sample were maintained.

Supplementary Table S6 shows the daily consumption of nutrients by the subjects in the upper quartiles. Compared with VNs and OMNs, FSs appeared to consume more fat, and VNs became the only group that differed in SFA consumption from the other groups. VNs of the upper quartile consumed protein at approximately the same level as OMNs did. The median n-3 PUFAs consumption in all the diets reached the recommended intake level. In addition, cholesterol consumption also increased in FSs. Only in VNs in the upper quartile the median cholesterol consumption did not exceed the recommended values. FSs and VNs consumed much more carbohydrate than other groups did, but their sources of carbohydrate were different. Thus, MDS consumption increased in VNs due to the predominance of fruits in their diet. The intake of cereals increased in FSs, which explains the increase in total carbohydrate due to starch. The amount of Ca significantly increased in all the plant-based groups. All groups in the upper quartile except VNs consumed an adequate amount of Se compared with the reference values for both genders. Additionally, the consumption of vitamin B_12_ in FSs almost equalled with the OMNs’ level, and in the diets of both groups in the upper quartile, an adequate amount of this vitamin was observed. For the rest, the patterns of the total sample were preserved. In the upper quartile, OMNs also had the highest risk of inadequate intake of n-6 PUFAs, fibre, Mg, and B-group vitamins (except for B_12_), but the n-6:n-3 ratio maintained the most optimal values. OMNs and FSs consumed the highest amount of Zn, while for LOVs, even in the upper quartile, the intake was inadequate.

Table 3 shows a comparison of the plant-based diet (by merging the VN, LOV, and FS groups) with the OMN diet. The OMN diet was a source of more protein. The consumption of SFAs, cholesterol, and n-3 PUFAs was greater in the OMN diet, while total PUFAs and n-6 PUFAs intakes were higher in the plant-based diet group. The n-6:n-3 ratio was 1.5–2 times greater among plant eaters, especially in men. OMNs consumed approximately 1.5 times fewer carbohydrate and more than 2 times less sugars and fibre than other subjects did.

The amounts of K, Mg, and Fe were 1.5 to 2 times higher in the plant-based group than in the ONM group. The former also consumed more Co, Mn, Cu, Mo, and Cr. Higher I intakes were observed in OMN men, with no significant difference between female groups. Se content was also higher in the OMN diet, and a difference in Zn consumption was not found. Adherents of the plant-based diets consumed vitamins B_1_, B_2_, PP, B_5_, B_6_, H, B_9_, C, and E in greater amounts than OMNs did. In contrast, the intakes of vitamins B_12_ and D were greater in the OMN diet. No difference in RE consumption was found.

Table 4 provides the analysis of daily nutrient intake by the median caloric intake for all subjects. The OMN diet was the richest in protein, while the other groups consumed its similar amounts to each other. MUFAs consumption in VNs appeared to be equal to that in LOVs and OMNs. The differences in n-6 PUFAs consumption between FSs and OMNs disappeared. The intake of sugars in VNs appeared to be greater than that in FSs. OMNs consumed the lowest amounts of both sugars and carbohydrate in general. The K consumption in FSs equalled to that in OMNs, whereas the Mg, Fe, and B_9_ intakes appeared the same as those in LOVs. With the same caloric value of diets, the major I and Zn consumers turned up to be OMNs, and the intake of these elements in FSs approximately equalled to that of VNs and LOVs. FSs consumed less Co and vitamin E than LOVs, and less Mn than all the groups. The amount of Cu in their diet became equal to that in the LOV diet. Compared with VNs, FSs consumed less vitamin B_1_, and B_2_—compared to all the other groups. The vitamin PP intake appeared to be approximately equal to that of the other groups (except for the LOVs). In other respects, the patterns of the calorie-unadjusted analysis were preserved.

The unadjusted correlation analysis revealed plenty of beneficial medium-weak linear associations (r = 0.3–0.5) between the adherence to any of the plant-based diets and nutrient intakes or calorie adjusted nutrient intakes. Particularly, plant-based diets directly correlated with fibre, K, Mg, Fe, Co, Cu, and vitamins B_1_, B_5_, B_6_, H, B_9_, C and E consumption, whereas reversely—with SFA and cholesterol intakes (for the last one, r = −655, *p* < 0.001). Two more medium-strong positive correlations (r > 0.5) were found between vitamins B_12_ and D intakes and the OMN diet. At the same time, most correlations became significantly weaker after adjustment by calorie intake (Supplementary Table S7).

After the gender, age, and BMI adjustment significant linear associations were observed for SFA, n-6 PUFAs, n-6:n-3 PUFAs ratio, cholesterol, total carbohydrate, MDS, fibre, K, Mg, Fe, Co, Cu, Se, and vitamins B_1_, B_6_, H, B_9_, B_12_, C, D, and E intakes (Supplementary Table S8). After calorie intake adjustment associations only for cholesterol and vitamins D and B_12_ remained. Gender and age were found to significantly affect Se intake, whereas “gender + BMI”, “age + BMI”, and “gender + age + BMI” associations impacted cholesterol consumption. The latter factor was also associated with SFA and vitamin D intake. In the calorie adjusted regime, “age + BMI” factor was found to impact cholesterol and vitamin D intake. No other association were found when excluding the effect of diet.

## 4. Discussion

This study investigated the nutrient intake of different plant-based dietary groups in a sample of the Moscow population. VNs and OMNs were more likely to have the largest differences in intakes, whereas LOVs and FSs were predominantly in an intermediate position.

### 4.1. Macronutrients

The regularities of calorie values in other studies differed. In some of them, the caloric value of the diet increased with an increase in the share of animal foods in it [57,69,70,71,72,73]. At the same time, according to Schüpbach et al., VNs had the highest total calorie intake, LOVs had the lowest calorie intake, and OMNs collocated in an intermediate position [74]. Bruns et al. [75] did not find a significant difference in the energy value between VNs and OMNs, and Blaurock et al. [76] between LOVs and OMNs. In a 2023 Spanish study, a difference in caloric intake between VNs, LOVs, and OMNs was also not present, while LOVs and OMNs had ~ 1.3 times greater caloric intake than our corresponding participants did [77]. In our recent Italian study, we also did not observe differences in calorie values between VNs, LOVs, and OMNs [78]. The calorie values of the FS diets in other studies were significantly lower than those in our study [39,42,79,80,81,82] and usually decreased during FS periods [83].

A number of studies supported our results on protein intake in VNs, LOVs, and OMNs [71,76,78,84,85,86,87,88,89], while FSs in our study consumed more protein than those reported by other authors [42,79,80,81,82,90]. In the Elorinne et al. study, OMNs consumed 1.5 times more protein than our subjects did [91]. Notably, VNs in the upper quartile equalled with OMNs in terms of protein intake. Rizzo et al. [92] found no differences between the groups when converted to total caloric intake. A systematic review by Koufakis et al. [93] showed that protein intake during the Lent can be either higher or lower than that associated with an OMN diet. However, even though all amino acids can be found in a plant-based diet [94,95,96], some observational data suggest that lysine [97,98] and sulphur-containing amino acids [97,99,100] could be critical, and this issue can arise from poorly balanced dietary habits. At the same time, Russian citizens [101], as well as people from East and South Asia [102], consume much more fermented foods, which can be a good source of essential amino acids [103].

In general, our results on fat intake were similar to those of other studies [74,76,86,87,91,93]. Ho-Pham et al. reported much lower fat consumption in VNs and OMNs [72,73]. In a recent Nepali study, fat intake among LOVs was almost twice as low as that among our LOV group [89]. OMNs, according to different data, consumed 1.3–1.5 times more fat than they did in our study [70,104], unlike the Baroni et al. study [78], where they consumed only 58 g of fat. In other studies, VNs consumed 1.4–1.7 times more fat [104,105]. In many studies, VNs consumed 1.5–2.6 times more SFAs than our subjects did [74,86,91,105]. According to García-Morant et al. [105], VNs consumed approximately 1.5 times more PUFAs than did VNs in our study. In contrast, in a German study, PUFAs intake in LOVs was almost two times lower than that in our subjects [76]. In a recent Italian study, all the groups consumed ~30% less PUFAs [78]. Rizzo et al. [92] reported no differences between the groups in standardizing diets to the total calorie value, which contrasts with our results. A 2021 systematic review [71] revealed the highest PUFAs intake adjusted to the caloric value in VNs, whereas the lowest PUFAs intake was in LOVs, which was the complete opposite of what we observed. Interestingly, Bakaloudi et al. [88] noted that the reported consumption of PUFAs increases significantly over time. The FSs in our study were expected to consume less fat, especially SFA [42,43,79,80,81,83,93], as both animal and plant fats (oils) were restricted during most Lent days. According to other studies, FSs consumed 4–5 times more MUFAs than our subjects did [79,80,82], and the results of Bloomer et al. [42] were similar to ours. All this information may indicate that today, total fat and fat fractions consumption is less dependent on the type of diet (i.e., VN, LOV, etc.) but more on the culture of additional fats intake, such as oils or butter, set in a particular region or subgroup of people.

The small amount of n-3 PUFAs in the VN and LOV diets in our study may be due to the absence of fish in the diet and poor seaweed intake, which are the main dietary sources of this class of substances. Interestingly, in a study by Allès et al. [86], the VN diet had the highest n-3 PUFAs content, followed by the LOV and OMN diets, which was supported later in a systematic review [71]. Blaurock et al. [76] reported no differences between LOVs and OMNs. However, VNs and LOVs consume n-3 PUFAs predominantly in the form of the precursor α-linolenic acid [22,106,107]. The main dietary source of long-chain n-3 PUFAs is oily fish [106]. α-Linolenic acid can be converted into eicosapentaenoic acid (EPA) endogenously, which, in turn, can be prolonged into docosahexaenoic acid (DHA), but the efficiency of such conversion is probably not very high; however, this issue lacks human studies. Rizzo et al. [92] reported that the intake of n-3 PUFAs did not differ between groups and exceeded the values of our subjects by 1.7–3.5 times. With respect to the intake of n-6 PUFAs, Allès et al. [86] supported our data. Knurick et al. [84] found no differences between the groups, with LOVs and VNs consuming 1.5 and 2.6 times less n-6 PUFAs than in our study, respectively. The moderate risk of n-6 PUFAs inadequacy in different groups and, especially in OMNs, indicates insufficient consumption of plant oils, nuts, and seeds. However, there could be an underreporting of added fat intake, particularly among OMNs. On the other hand, the n-6:n-3 PUFAs ratio seems even more important than the absolute PUFAs intake levels [108]. The n-6:n-3 ratio in FSs in our study was comparable to that reported by Papadaki et al. [79] and Kokkinopoulou et al. [82], whereas Sarri et al. [39] found this ratio for FSs and OMNs to be 11.5 ± 0.6 and 13.0 ± 0.6, respectively. The n-6:n-3 ratio distribution features through the quartiles indicate that VNs ought to strive shifting it towards n-3 PUFAs not only by regularly consuming the latter, especially EPA and DHA, but also by controlling n-6 PUFAs intake via adjusting plant oils use.

The same patterns of cholesterol intake were reported in other studies [57,69,74,76,77,78,80,81,82,86]. In a number of studies, VNs consumed 6–40 times more cholesterol than our subjects did [57,69,86,91,105]. Notably, cholesterol intake was normal in the lower quartile of the OMN diet. Sarri et al. [39] reported a 1.8-fold lower cholesterol consumption in their OMNs than in our subjects did, whereas Baroni et al. [78] reported a 2.7-fold lower cholesterol consumption. Considering the high risk of cholesterol excess in the OMN diet, the importance of fibre consumption is increasing, as it prevents the absorption of excess cholesterol in the gastrointestinal tract [109]. In this case, maintenance of tocopherol:cholesterol level is also important, as vitamin E plays a significant role in preventing the oxidation of serum lipoproteins [110]. We should note, that ideally, vegans may not consume cholesterol at all, as they totally avoid animal-derived products, which only contain cholesterol. However, various at the first sight vegan products contain hidden animal components, predominantly fats, as eggs, milk, or butter, are regularly used in cooking. Hence, vegans are often unaware that they consume non-vegan food. Probably, some of them ignore this fact due to trace amounts of animal-derived components in a product. Another source for such results may be the non-endless choice of foods in the FFQ, which sometimes lacks vegan options of various products and, thus, some vegan foods were calculated as non-vegan.

Our data on carbohydrate consumption are in line with those of other studies [89,91,105]. Allès et al. [86] reported similar patterns, but the VNs in their study consumed 1.5 times fewer carbohydrate. At the same time, in a recent study by Kokkinopoulou et al. [82], FSs consumed only 160 g of carbohydrate per day. According to Schüpbach et al. [74], VNs also consumed the largest amounts of carbohydrate in general, as well as sugars in particular, but there were no differences between LOVs and OMNs. In other studies, no differences were found between the dietary groups [69,78,85,87,92,111]. The patterns of fibre consumption that we found were confirmed in other studies [23,43,57,69,70,71,74,76,77,78,86,87,88,91,105,112,113]. Nevertheless, VNs in other studies [23,42,69,75,78,86,87,91] consumed approximately 1.5–2 times less fibre than did those in our research. According to a systematic review by Koufakis et al. [93], the consumption of carbohydrate in FSs can be either higher or lower than that in the OMN diet. Literature reviews also showed that during the Lent, the consumption of fibre is greater than that of a regular diet [43,83,93]. Importantly, even in the upper quartile, OMNs still consumed a critically low amount of fibre in our study, which indicates that this group needs to monitor the consumption of fibre and increase the proportions of vegetables, greens, and whole grains in their diet. At the same time, the recent paper by Kokkinopoulou et al. [82] revealed no difference in fibre intake between FSs and OMNs, while the mean value was only 21 g/day.

Interestingly, our previous study did not reveal any differences in macronutrient composition of the diets of students from many regions of the world, even when compared with most other studies [114]. However, this was true only for students from cities, but not from rural areas. Subjects from countryside in assessments of Chinese [115] and Brazil [116] authors consumed less fat and more carbohydrate.

### 4.2. Macro Elements

K consumption in the Sobiecki et al. [57] study did not differ between VNs and OMNs, while intake in LOVs was lower. Blaurock et al. [76] did not find any differences between LOVs and OMNs. The predominance of K in VNs has been shown in many studies, which is explained by its high content in fruits and vegetables [70,78,85,86,105,117]. Our data among FSs are confirmed in studies by Papadaki et al. [79], Karras et al. [81], and Sarri et al. [41].

As expected, LOVs were the major Ca consumers because of the high proportion of dairy in their diet. Thus, in a study by Sobiecki et al. [57], LOVs consumed the highest amounts of Ca, but the lowest intake was observed among VNs. Similar values and regularities have been reported in other studies [71,74,76,86]. Our recent Italian study did not reveal differences between VNs, LOVs, and OMNs [78]. In Clarys et al. study [69], the calorie adjusted data were similar to ours except that OMNs consumed more Ca than VNs did. A 2023 meta-analysis [118] revealed lower Ca intake in VNs than in LOVs but no difference between LOVs and OMNs. According to the literature reviews [43,83], during the Lent people consume less Ca than do those fed an OMN diet. In addition, the situation for VNs and FSs is exacerbated by the fact that Ca in plant products often has significantly lower bioavailability because of the action of absorption inhibitors, such as oxalic and phytic acids [24]. However, leafy vegetables with low contents of these substances are not inferior to or even greater than dairy products in terms of both the content and bioavailability of calcium [119,120,121].

The same particularities of Mg consumption in different groups were revealed in other studies [57,71,74,75,76,79,85,86,105,113,117]. At the same time, in the Knurick et al. [84] study, LOVs and VNs consumed 1.7 and 1.9 times less Mg than in our study, respectively. Baroni et al. [78] did not find any differences between VNs, LOVs, and OMNs, while VNs consumed 1.9 times less Mg than our subjects did. Sarri et al. [41] reported no differences between FSs and OMNs, with the former consuming approximately 1.5 times more Mg in our study than in this one. Mg deficiency in the OMN diet in western societies is common, and due to the high Mg content in herbs, nuts and seeds, its consumption increases with an increase in the proportion of plant foods in the diet [122,123,124]. The Mg consumption in FSs in the Karras et al. [81] study was lower than our results, even compared with data converted to lower calorie levels.

P intake by different groups has generally been confirmed in other studies [71,74,78,79,85,86,92,105,113]. VNs (in Ströhle et al. study [117]) and OMNs (in Lindqvist et al. study [70]) consumed 1.4 and 1.6 times more P, respectively, than our subjects did. The same was shown for LOVs by German authors [76]. In general, P is not an element of concern for any of the groups [124].

### 4.3. Trace and Ultratrace Elements

Similar patterns of Fe consumption were observed in other studies [57,74,75,78,88,105,113]. According to Śliwińska et al. [125], the largest amount of Fe was consumed by women in the VN group. In most studies, VNs consumed 1.6–2.4 times less Fe than our subjects did [70,71,78,85,86,91]. A high Fe content in the VN diet was quite expected, as plant products are rich in this element [126]. A recent review of FS diets also supported this finding [83]. However, the average bioavailability of Fe from plant products is usually lower and is approximately 10% [127,128].

In a study by Sobiecki et al. [57], VNs consumed less I than LOVs did, and OMNs were the major consumers. The same pattern was shown in two systematic reviews [71,88]. German authors did not find any difference between LOVs and OMNs [76]. However, the I intake in all the groups was 1.5–3 times greater than that in our study. A systematic review conducted in 2020 concluded that VNs and LOVs, which do not consume algae or I-containing supplements, have a greater risk of inadequacy for I [129]. A 2023 review confirmed that I intake remains a major concern for VNs, as on average, they consume only 1/3 of the recommended value [130]. Despite these differences, all the groups are at serious risk of I deficiency in continental regions with poor soil I contents, such as the European territory of Russia [131]. An effective way to combat I deficiency is to consume iodized salt [132] and to use dietary supplements and more sea algae. Nicol et al. [130] also suggest an implication of wide governmental programs of food fortification with I.

Hokin et al. [133] reported similar data on Co intake in VNs and LOVs, but with the exclusion of two VN subjects from our study who consumed large quantities of potatoes, the results of VNs and LOVs were similar. OMNs consumed Co the most, with values 3.2 times higher than our data. In general, little is known about Co deficiency [134]. The Mn consumption data were similar to the data from Allès et al. [86], and the Cu intake data were the same as those from Sobiecki et al. [57] and Allès et al. [86]. Importantly, phytates do not prevent Cu absorption in the stomach [128], but the bioavailability of Cu in plant-based diets is still lower than that in OMN diets [135]. This may be due to the negative impact of ascorbic acid, which reduces Cu^2+^ to Cu^+^, preventing its absorption, as well as due to the competition for divalent metal transporter-1 (DMT-1), which absorbs divalent metals, with iron, which is present in larger quantities in the VN and LOV diets.

We found no data describing Mo and Cr consumption in different types of diets. Although, the intake of these elements was significantly higher in VNs and LOVs than in OMNs and, especially, FSs, Mo and Cr intake inadequacies were almost totally spread. The richest in Mo product is fish caviar. It also can be found in other fish tissues and seaweed, animal liver, legumes and whole grain products, dark leaves, and currant berries. The main sources of Cr are fruits and seafood [58,136]. Considering our results and very poor knowledge on the status of these elements in population we believe, that further research is required.

In a study by Sobiecki et al. [57], VNs consumed more Se than LOVs did, which contrasts with our results. Blaurock et al. [76] reported no differences between LOVs and OMNs; moreover, the Se intake levels in both groups were extremely low: 4 µg and 7 µg, respectively. Notably, the level of Se in plant foods directly depends on its content in soils [137]. In many regions of the Russian Federation, there are biogeochemical provinces with Se deficiency [138,139,140]. Moreover, Se is less available for plants from acidic soils [141,142], abundant in Eastern Europe [143]. All these justifies the use of selenium-containing fertilizers. The high content of Se in fish and seafood provides OMNs and FSs with this element, and some of its content in eggs [144] may be a good source for LOV. At the same time, Brazil nuts could be a good source of Se for all the groups [134]; however, their high cost in Russia diminishes their consumption.

Papadaki et al. [79], Allès et al. [86], and Knurick et al. [84] observed similar patterns of Zn consumption in different groups. In the Rizzo et al. [92] study, Zn intake was 1.4–1.9 times higher than that in our subjects in all groups when it was converted to the median caloric value. Blaurock et al. [76] calculated even two times higher Zn intake in LOVs. In the 2013 meta-analysis, Zn intake was significantly lower in vegetarians than in the control groups [26]. In a recent study [145], Zn intake in VNs was quite equal to that in our VN group, whereas OMNs consumed ~ 1,5 times more Zn than OMNs did in our study. Another 2023 study reported no differences in Zn intake between VNs and OMNs [75]. In Baroni et al. study [78], all the groups consumed 1.1–1.4 times more Zn, and no differences were found. A 2021 systematic review revealed no difference in Zn intake between VNs, LOVs, and OMNs [71]. Zn is found both in plant products (leafy vegetables, seeds and nuts) and in meat and seafood [144]. Although Zn intake was comparable between our groups, the fact that the bioavailability of Zn decreases in the presence of phytates is important [144], so the recommended Zn intake rates for VNs and LOVs can be up to 50% higher [146].

### 4.4. Water-Soluble Vitamins

Many studies support our findings on vitamin B_1_ consumption [57,71,74,76,78,79,85]. OMNs in the Sarri et al. [39] study consumed 1.9 times less vitamin B_1_ than our subjects did, and the FSs results were similar to ours, with no significant differences between groups, which is in line with the results of Koufakis et al. systematic review [93].

In many studies, the greatest amount of vitamin B_2_ was found in the OMN diet [57,70,76,78,85,86,87,88]. However, a 2021 systematic review revealed no differences between dietary groups [71]. Thus, our results stand out from the general data. The results of FSs were comparable with those of other studies [79,81]. According to Sarri et al. [39], OMNs consume more vitamin B_2_ than FSs do, which is consistent with our results converted to the total energy value.

In the studies by Sobiecki et al. [57], Allès et al. [86], and Schüpbach et al. [74], LOVs had the lowest vitamin PP intake, which was later supported in a systematic review [71]. Blaurock et al. [76] observed no difference in niacin intake between LOVs and OMNs, while Baroni et al. [78]—between VNs, LOVs, and OMNs. In the Kristensen et al. [85] study, OMNs consumed 1.8–2.6 times more niacin than did OMNs in our study. In addition to the food niacin, endogenous synthesis from the amino acid tryptophan may also be its source [147]. A systematic review by Koufakis et al. [93] showed that niacin consumption does not change significantly when the Lent is adhered.

Our results concerning vitamin B_5_ intake are mostly in line with previous studies [74,87]. The FSs’ intake of pantothenic acid in the Papadaki et al. [79] study was approximately 1.5 times lower than our findings. VNs in our study showed the highest consumption of vitamin B_5_, as well as B_6_, even in terms of median calorie intake. Rizzo et al. [92] revealed that OMNs and LOVs consumed approximately twice as much vitamin B_6_ as our subjects did. Blaurock et al. [76] reported no differences in vitamins B_5_ and B_6_ intake between LOVs and OMNs, but the values were up to 35% greater, especially for pantothenic acid. Our results are in line with those of Neufingerl et Eilander [71] systematic review, which revealed the highest vitamin B_6_ intake in VNs.

We did not find sufficient data on biotin intake by different groups. The predominance of biotin in our study in VNs can be explained by the fact that its main sources are legumes and grains [58]. All the groups had a high risk of inadequacy in its consumption, regardless of diet. This is likely largely because products lose the greatest amount of biotin, as well as many other nutrients, during refinement [148]. Moreover, the biotin status of the population has not yet been fully characterized. Blaurock et al. [76] reported an order of magnitude greater value for vitamin H intake and no difference between the LOV and OMN groups.

Several studies supported our findings in folate consumption [74,78,86], and some data almost completely coincided with our results [76,79,84,85,104], including relative values [39]. OMNs in some studies consumed approximately 1.5 times more folate than our subjects did [42,78,91]. According to reviews by Lazarou et al. [43] and Giaginis et al. [83], FSs also consume more folate than OMNs do. Despite the fact that the risk of folate inadequacy was expected, LOVs had a surprisingly high risk of inadequacy of this nutrient compared with most studies [71], which may indicate insufficient consumption of leafy vegetables in the Russian LOV population.

In almost all studies that evaluated the intake of vitamin B_12_, the values for all groups were significantly higher than those we obtained [57,70,71,74,75,76,84,86,87,91]; however, our recent Italian findings [78] appeared to be very close to what we observed in Russia in the current study. The relative intake level data from Rizzo et al. [92] were also significantly greater. In the upper quartile of FSs, cobalamin intake reached normal values due to an increase in the consumption of marine invertebrates. It is critically difficult for VNs to consume enough vitamin B_12_ because plants do not synthesize or accumulate it.

It is worth noting that, although, there is some evidence of lower frequency of an unfavorable MTHFR gene variation (rs 1,801,133 T/T genotype), which reduces the efficiency of the folate cycle and, thus, increases the need for vitamins B_2_, B_6_, B_9_, and B_12_ involved in it, in Russia [149], than in most other regions [150,151,152], these data cannot be considered as reliable, as most data contain the spread of MTHFR polymorphisms in affected populations. Large-scale investigations in healthy subjects are needed to make reliable conclusions in this regard.

VNs’ intake of vitamin C in our study exceeded the results of many authors by 1.4–3 times [70,71,74,78,84,85,86,87,91,105,117] and LOVs’—by 1.4–2 times [70,76,78,84,86,87,113]. OMNs, in the studies of Elorinne et al. [91] and Rizzo et al. [92], consumed 1.8–1.9 times less vitamin C than our subjects did. The data from our FS subjects were comparable with those of other studies [42,79,81].

### 4.5. Fat-Soluble Vitamins

The data on RE consumption vary among different sources in the literature. Our results for VNs and OMNs were supported by García-Morant et al. [105], and those for FSs by Bloomer et al. [42]. German authors [76] also reported no difference in RE intake between LOVs and OMNs, but the values were 70% and 60% greater, respectively. Baroni et al. [78] also revealed no differences—LOVs and OMNs in that study consumed much more RE, whereas VNs consumed approximately the same amount. Vitamin A intake in FSs in the Karras et al. [81] study, despite lower caloric intake, was 1.5–1.7 times higher than that in our subjects, and in other studies it was even 1.6–2 times higher [39,79]. We suppose that this difference may be explained by the greater availability of fruits in Greece, where the above studies were conducted, than in middle Russia during the Lent period. Indeed, it is usually snowy in Moscow in April. The same reason determined the lag of FSs from the other groups in RE intake: the absence of seasonal fruits in the diet analysis sharply reduced the intake of carotenoids. At the same time, a systematic review by Koufakis et al. [93] revealed that vitamin A intake decreased during the Lent compared to OMN diet. Despite the poor retinol content in the diet of VNs, the large variety of carotenoids in their diet provides a fairly high total intake of RE [153], and a 2021 systematic review revealed no differences in RE intake between VNs, LOVs, and OMNs [71].

Similar to ours peculiarities of vitamin D intake were systematically observed [57,70,71,74,86,87,88,91,105], but the absolute values were usually higher for all groups. Blaurock et al. [76] did not report any differences in vitamin D intake between LOVs and OMNs. However, in all the studies, most subjects still had a high risk of vitamin D deficiency. Decreased 25(OH)D levels are widespread, regardless of diet type [154]. Particularly, adequate serum calcidiol concentrations were observed in 211 out of 818 subjects (25.8%) in West Siberia (Omsk, Russia, 55–56° N) [155]. 25(OH)D levels in 32.5% of subjects corresponded to insufficiency (20—30 ng/mL) and in 41.4%-to deficiency (<20 ng/mL [156,157]). Median calcidiol level was only 22.2 ng/mL with the annual variation between 18.7 ng/mL in January and 26.5 ng/mL in October. Among Saint-Petersburg (Russia, 59–60° N) adults, mean serum 25(OH)D concentrations were 21.9 ng/mL with 34.2% of subjects with insufficiency and 47.9%—with deficiency, whereas in Petrozavodsk (Russia, 61–62° N) mean calcidiol levels were 19.8 ng/mL and only 8.4% were adequately supplied with vitamin D [158]. Vegans in Moscow (Russia, 55–56° N) had similar serum 25(OHD) concentrations, even those, who took vitamin D supplements (22.5 vs. 20.7 ng/mL, respectively) [159]. The situation in the USA or Europe, even South Europe, is not different drastically but may be even worse in Middle East and South Asia [160], probably, due to higher skin pigmentation and culturally determined dress codes.

At the same time, one should be careful with uncontrolled vitamin D supplementation, as excess of vitamin D, as well as the other fat-soluble vitamins, can be not less harmful [161]. Moreover, the vitamin D status turns out to be more a lifestyle issue than a nutritional one, despite the word “vitamin” in it. The prevalence of vitamin D deficiency increases with increasing latitude, as endogenous synthesis of calciferol becomes impossible under low insolation [162]. Cutaneous calciferol synthesis may also be impaired due to due to the prevalence of cloudy days or atmospheric pollution with dust or industrial emissions [163], which is becoming a pressing problem in many regions of the Global South, among others [164,165,166]. Reduced calcidiol serum concentrations may be a factor contributing to impaired Ca metabolism, which requires an extra attention from the plant-based diets adherents [167].

The pattern of vitamin E intake in our subjects was similar to that reported in most other studies [57,71,74,85,105] but was 1.1–1.8 times greater. In our study, even when converted to lower calorie intake, FSs consumed approximately 1.5–3 times as much vitamin E as in other studies [39,42,79,81]. This may be due to the specificity of Russian culinary traditions, namely the overwhelming dominance of sunflower oil, which is richer in tocopherol content than the majority of other oils [168].

### 4.6. General Considerations

All groups to varying degrees consumed inadequate amounts of n-3 PUFAs, Ca, I, Cr, Mo, Zn, as well as vitamins B_2_, PP, H, B_12_, and D. We labelled these nutrients as “risky nutrients” (RN). Given the 100% risk of vitamin D inadequacy for all groups, it is a special cause for concern. The same concerns, but to a lesser extent, were risen regarding vitamin B_12_, especially for VNs and LOVs, but luckily the problem is well-known and these dietary groups are used to supplement it and regularly monitor the level of cobalamin and homocysteine in the body. It is also recommended for these two groups to control the I status. In contrast to cobalamin, it is possible to increase the consumption of I by regularly using seaweed and iodized salt.

VNs had the highest consumption of fibre, Mg, folate, vitamin C, both in total and lower and upper quartiles. In the VN group, Se was the only addition to RN.

LOVs had significantly greater risks of Mg and folate inadequacy. Also, among them, there was often observed a decrease in the intake of Se, vitamins A, B_1_, B_5_, and B_6_, in addition to the RN group. In general, for LOVs an additional intake of B vitamins may be recommended. 2/3 of LOVs and 3/4 of VNs had and increased n-6:n-3 PUFAs ratio in the diet, which also requires a special attention from these groups.

In our study, in addition to RN, FSs were particularly characterized by insufficient intake of Mg, vitamins A, B_5_, B_6_, and folate. Despite the fact that the Lent is a transitory diet, it is still advisable to ensure that the diet is varied and balanced.

It is interesting that OMNs, having the widest range of products allowed to eat, had an inadequate consumption of the largest number of nutrients. In addition to RN, OMNs should pay attention to compensation of fibre, K, Mg, Fe (especially in women), as well as vitamins A, B_1_, B_5_, B_6_, B_9,_ and E in the diet. Cholesterol was another RN for OMNs, as 81% of them consumed it excessively.

Thus, in our study, the VN diet was more adequate for many nutrients, in comparison to OMN diet. This was especially true for fibre, K, Mg, Fe, vitamins A, C, E, and many B vitamins (except B_12_). LOVs and FSs were in many ways in an intermediate position.

In general, plant-based diets adherents consumed less SFA, cholesterol and more K, Mg, Fe, Cu, vitamins B_1_, H, B_9_, and E. On the other hand, OMN diets contained more Se and vitamins D and B_12_. Additionally, plant-based diets had significantly higher total carbohydrate content. Among VNs, MDS consumption often was significantly higher than the recommended upper limits. Noteworthy, comparison with previous studies showed that the diet tends to be more nutritious and thoughtful among Russian VNs than among VNs in most other regions of the world. However, such a comparison inevitably is a source of bias due to the variations in study methods (FFQ or dietary recall, period analysed, differences in bias of specific tools and techniques, etc.), thus, should be considered with caution. Another important thing to mention is the unexpected low energy intake in OMNs, taking into account their BMI values. The most obvious reason for that is the potentially lower physical activity in the OMN group. However, we did not assess the physical activity in our subjects. At the same time, an intake underreporting could take place in this group. If so, OMNs might have had higher absolute intakes, lower deficiencies frequency, and even a more severe excess of cholesterol in their diet, but with no changes to calorie adjusted nutrient intake. Moreover, underreporting could also be product-specific, i.e., “junk food” could be underreported with an adequate reflection of other products consumption. Then the OMN diet would appear more caloric, with higher macronutrient content, especially fats, but with further decrease in micronutrient density. Other probable reasons for such discrepancy could be medical obstacles (hyperthyroidism, maldigestion, malabsorption, parasite invasion, oncology, etc.), however, we did not include subjects with clinically manifested diseases in this study, whether diagnosed.

Despite the predominance of many beneficial nutrients in plant-based diets, it is necessary to keep in mind the existence of antinutrients, such as phytic and oxalic acids or tannins, which are abundant in plant foods and can significantly reduce the absorption of some micronutrients, especially, divalent metals, such as calcium, magnesium, manganese, iron, copper, or zinc. In addition, some nutrients in plant foods are in a less absorbable form. These facts may lead to an increased need for particular micronutrients in plant-based diets adherents and justify further studies aimed to creation adjusted for various plant-based diets recommended intake levels [124,128].

### 4.7. Strengths and Limitations

We could not find any previous studies comparing the nutrient contents of VN, LOV, and the Christian Lent diets. In addition, a wide list of nutrients was evaluated. Finally, the geography of this study is another strength, as data on vegetarians, as well as FSs’ nutritional status, are extremely limited in this part of the world.

Unfortunately, this study has several limitations. First, a limitation of the study was the method of diet assessment itself. It was suggested that FFQ method somewhat averages fat intake [169]. At the same time, an underreporting of added fat intake could occur, especially in OMNs, who are usually less aware of what they eat [170,171,172]. On the other hand, an overreporting is more typical for the FFQ method [173], while the underreporting—for dietary recall [174].

The analysis of nutritional anamnesis by the FFQ method is based on the recollections of patients, and it is imperfect as much as the mnestic apparatus of the studied people is imperfect. In this context, the diary method, in which the subjects record their diet prospectively, is preferred. However, given that we attempted to assess nutrition for the entire year (with the exception of the FS group, which was assessed only during the Great Lent), the diary method was inapplicable, since it seems very problematic to convince the study group to keep a nutritional diary throughout the year, and the dropout rate would be enormous. It would be irrational to offer subjects to record their food for a week or even a month, since nutrition can undergo significant seasonal changes, and, for example, summer fruits and berries could not get into the diary recorded in winter. A possible solution could be a significant enlargement of the sample size provided with an equal spread of the subjects throughout all the year, however, such a study would have required much more resources than were available for us, while such design would still have a significant bias due to separating subjects through exact seasons without any possibility to see the subjects’ annual diets as a whole. Furthermore, vegans in Moscow are “ones of a kind” and it would be rather difficult to recruit a larger sample size. Moreover, the FFQ method is supposed to rely more on the subjects’ habits, rather than on their true recall accuracy. At the same time, it should be pointed out that till now there is no fully reliable method for holistic assessment of the diet, and the preferable approach is a subject of hot debates in nutritional science [175].

We believe, it is also important to note, that FFQ is not a single method but a group of approaches. And the name “Food frequency questionnaire” most precisely reflects only the simplest versions of it, where paper questionnaires often even without exact portions are given to the studied subjects to fill in at home. Modern and most reliable variants of FFQ include large row of products and dishes and life-size photos of them, whereas the “questionnaire” is filled in specialized software with assistance of professional dietician [176].

As fortified foods were not included in the Nutrilogic database, these products were calculated as not fortified. Hence, the true intake of some micronutrients in the subjects could be slightly higher. However, we believe this limitation did not shift our results significantly, due to very poor availability of fortified foods in Russian markets. Supplements use was also not considered in our calculations as our aim was to assess the diets themselves. We have discussed this limitation in detail in the Appendix A. Due to limited resources no validations with food diary or biomarkers were made to minimise the dietary data collection bias. Furthermore, as any other nutrition assessment method does, FFQ fully relies on subjects’ self-reporting without any robust objective ways to control their testimonies.

The mentioned above quite poor sample size of our study is another limitation, which, unfortunately, reduced its impact. Particularly, we did not divide subjects inside groups into dietary patterns, such as “orthorex”, “fast-food”, “cook of the house”, etc. In addition, as the OMN group was formed based on the anthropometric parameters of other three groups, the subjects’ selection cannot be fully considered as randomized.

Our statistical analysis also had several flaws. We could not balance our study for a four-way analysis of variance with four levels in diet parameter. Thus, a two-way (diet and age) and three-way (diet, sex, and BMI) analyses of variance were performed. During the four-way Yates’ analysis with two levels for diet parameter the impact of the “diet + age” association could not be calculated because of the technical features of the analysis and, finally, because the study was not very well balanced. We should also note that our study had quite a high multiple regression shift coefficient. This means that there was a significant risk for false-negative results. At the same time, the differences that still were found are very unlikely to be false-positive.

## 5. Conclusions

The main trend revealed was an increase in the micronutrient density when switching from a more animal-based to a more plant-based diet. The opposite was observed only for selenium and vitamins B_12_ and D, however, the consumption of the latter was ~15 times less than the estimated adequate intake even in omnivores. Plant-based diets also demonstrated a more beneficial macronutrient composition, especially with regard to fibre and lipids, except for n-3 PUFAs. Some micronutrients were within a high risk of inadequacy in all the diet groups. In addition to calciferol, those were Ca, I, Cr, Mo, Zn, vitamins B_2_, PP, H, and B_12_.

All the studied groups require significant improving of their diets in various aspects. This could be achieved through improvement and expansion of nutritional education of the population and especially healthcare sector specialists. Another crucial thing is the lack of food variability in all the groups, which, among others, requires a better availability of certain products rich in nutrients that are in scarce supply.

We hope our results will be useful for future studies in plant-based nutrition and, taking into account quite poor nutrition of the OMN group, in public nutrition in general. We also believe our research could be of importance for education of specialists in public health, nutrition epidemiology, clinical practitioners, and, of course, for nutritionists, dieticians, and caterers.

## Figures and Tables

**Table 1:** Daily dietary nutrient intake in different nutritional groups, Me (25th percentile; 75th percentile), unadjusted model.

Intake/Day *Significance*	VN	LOV	FS	OMN	Reference Values/Units
**Energy value** *cdF*	2190 (1729; 2676)	1826 (1441; 2618) *	2462 (1911; 3290) *	1774 (1537; 1946)	kcal
**Protein** b*cde*	58 (46; 69)	56 (43; 72) *	76 (51; 93)	70 (59; 74)	g
**Fat** c	64 (46; 96)	82 (57; 103) *	67 (54; 99)	82 (67; 92)	g
**SFA** *ABCeF*	9.0 (6.3; 14.4)	24.3 (15.7; 27.2) *	17.1 (9.8; 27.3)	29.1 (23.6; 32.0)	g
**MUFA** ac	17.2 (11.5; 23.2)	12.9 (7.7; 20.0)	12.2 (6.4; 23.8)	12.5 (11.5; 15.2)	g
**PUFA** ce	19.7 (11.3; 33.9)	27.3 (13.1; 31.7) *	19.8 (11.0; 30.4)	16.5 (12.6; 25.1)	g
**n-3** *BCDE*	0.6 (0.4; 0.9)	0.6 (0.5; 0.8)	1.3 (0.9; 2.2)	1.1 (0.9; 1.5)	1–3 g [67]
**n-6** *C*f	10.4 (6.2; 17.6)	8.9 (4.8; 13.7)	8.9 (4.8; 16.6)	6.5 (5.7; 7.7)	10 g [67]
**n-6/n-3 ratio** *BCDE*	18.0 (10.0; 27.2)	12.8 (7.8; 17.3)	6.4 (4.2; 9.6)	6.0 (4.8; 7.4)	5–10 [66]
**Cholesterol** *ABCdEF*	4 (2; 8)	188 (60; 258)	70 (23; 132)	404 (363; 505)	<300 mg [66]
**Carbohydrate** *ACDEF*	341 (272; 501)	245 (183; 348)	406 (314; 518) *	183 (114; 234)	g
**MDS** *AC*d*EF*	156 (126; 249)	125 (89; 145)	157 (105; 208)	65 (51; 97)	<75 g [67]
**Fibre** *ABCdEF*	60 (42; 74)	31 (25; 43)	41 (33; 54)	17 (15; 20)	RF—20 g [66] USA—25 g (f)/38 g [68]
**K** *ABCEF*	7069 (5384; 8640)	3732 (3164; 4755)	3861 (3169; 5253)	2635 (2310; 3243)	3500 mg [66]
**Ca** de	776 (587; 1053)	863 (694; 1133)	621 (509; 867)	736 (639; 842)	1000 (1200 –older 60) mg [66]
**Mg** *AbC*d*EF*	673 (467; 927)	401 (302; 557)	477 (378; 603)	288 (263; 328)	420 mg [66]
**P** f	1125 (851; 1444)	1086 (908; 1436) *	1234 (987; 1585)	1097 (960; 1234)	700 mg [66]
**Fe** *ABC*d*EF*	33 (24; 44)	20 (17; 27)	24 (18; 31)	16 (13; 17)	18 mg (f) 10 mg (m) [66]
**I** *adE*	71 (41; 102)	53 (34; 63) *	82 (36; 169)	72 (65; 87)	150 µg [66]
**Co** *ABCe*f	30.1 (23.4; 41.6)	18.4 (11.5; 25.3)	18.7 (9.3; 26.4)	12.3 (10.8; 16.9)	10 µg [66]
**Mn** *c*	6.4 (4.5; 8.7)	5.4 (3.3; 8.5)	5.0 (3.9; 7.3)	4.5 (3.6; 6.8)	2 mg [66]
**Cu** *AC*d*EF*	2.4 (1.9; 3.0)	1.6 (1.2; 2.4)	2.0 (1.5; 2.8)	1.1 (0.9; 1.3)	1 mg [66]
**Mo** *ABCDE*	39 (28; 63)	26 (18; 38)	14 (7; 25)	18 (13; 22)	70 µg [66]
**Se** *ABCDE*	25 (12; 38)	44 (28; 62)	64 (53; 109)	78 (65; 90)	55 (f)/70 (m) µg [66]
**Cr** *ABCDE*	31.7 (21.3; 45.1)	20.6 (14.6; 27.4)	10.8 (5.8; 17.7)	13.6 (10.3; 15.7) *	40 µg [66]
**Zn** a*DE*	7.5 (5.5; 9.0)	5.8 (4.2; 8.0)	8.3 (5.8; 11.9)	7.6 (6.7; 8.4)	12 mg [66]
**B_1_** *ACDEF*	2.2 (1.8; 2.7)	1.4 (1.1; 2.0)	2.2 (1.7; 2.8)	1.1 (0.9; 1.2)	1.5 mg [66]
**B_2_** c	1.7 (1.2; 2.3)	1.6 (1.1; 1.9) *	1.6 (1.3; 2)	1.4 (1.2; 1.6)	1.8 mg [66]
**PP (B_3_, niacin)** *ACD*e*F*	17.9 (14.8; 24.5)	12.4 (10.0; 17.9)	19.9 (16.5; 25)	14.5 (13.0; 16.8)	20 mg [66]
**B_5_** *AbCdF*	7.6 (5.2; 8.9)	3.8 (3.4; 5.3)	4.7 (3.7; 7.2)	3.8 (3.1; 4.3)	5 mg [66]
**B_6_** *ABC*e*F*	3.4 (2.2; 4.3)	1.7 (1.2; 2.2)	1.8 (1.4; 2.3)	1.4 (1.2; 1.6)	2 mg [66]
**H (B_7_, biotin)** *AbCEF*	15.7 (10.2; 25.3)	6.9 (5.1; 11.3)	9.9 (5.4; 18.2)	3.5 (2.9; 4.2)	50 µg [66]
**B_9_ (folate)** *ACdEF*	509 (363; 596)	332 (256; 499)	418 (316; 557)	252 (196; 285)	400 µg [66]
**B_12_** *ABCEF*	0.0 (0.0; 0.01)	0.3 (0.1; 0.6)	0.3 (0.0; 1.3)	2.3 (1.9; 3.4)	3 µg [66]
**C** *ABCEf*	430 (326; 572)	205 (142; 279)	157 (109; 249)	127 (82; 153)	100 mg [66]
**A (RE)** *aBCdf*	1141 (766; 1571)	767 (587; 917)	479 (307; 833)	722 (558; 871)	800 (f)/900 (m) µg RE [66]
**D** *ABCDEF*	0.0 (0.0; 0.0)	0.3 (0.1; 0.7)	0.0 (0.0; 0.2)	0.9 (0.5; 1.1)	15 (20—older 65) µg [66]
**E (TE)** b*CEF*	28 (19; 36)	23 (14; 34) *	22 (14; 30)	12 (9; 17)	15 mg [66]

VN—vegans; LOV—lacto-ovo-vegetarians; FS—fasters; OMN—omnivores; BMI—body mass index; SFA—saturated fatty acids; MUFA—mono-unsaturated fatty acids; PUFA—poly-unsaturated fatty acids; MDS—mono- and disaccharides; RE—retinol equivalents; TE—tocopherol equivalents. The values accompanied by * had a normal distribution; the means ± SDs were as follows: LOV (energy value: 1919 ± 967, protein: 73 ± 31, fat: 89 ± 54, SFA: 33.2 ± 25.5, PUFA: 18.1 ± 9.1, P: 1161 ± 497, I: 86 ± 50, B_2_: 1.5 ± 0.6, E: 14 ± 7), FS (energy value: 2606 ± 970, carbohydrate: 413 ± 143), and OMN (Cr: 13.4 ± 5.5). Comparison of peach-colored cells was performed via Student’s t test. A—*p* < 0.001 between VN and LOV, a—*p* < 0.05; B—*p* < 0.001 between VN and FS, b—*p* < 0.05; C—*p* < 0.001 between VN and OMN, c—*p* < 0.05; D—*p* < 0.001 between LOV and FS, d—*p* < 0.05; E—*p* < 0.001 between LOV and OMN, e—*p* < 0.05; F—*p* < 0.001 between FS and OMN, f—*p* < 0.05; *italics*—*p* < 0.05 after Holm-Bonferroni correction.

**Table 2:** Prevalence of inadequate dietary nutrient intake in different nutritional groups, % (n).

Intake/day *Significance*	VN	LOV	FS	OMN	Reference Values/Units
**MDS (excess)** *CEF*	97 (44)	84 (41)	88 (37)	16 (33)	<75 g [67]
**Cholesterol (excess)** a*CEF*	0 (0)	16 (8)	12 (5)	81 (39)	<300 mg [66]
**n-3** *BCDE*	85 (39)	86 (42)	42 (18)	40 (19)	1–3 g [67]
**n-6** *C*e*f*	46 (21)	65 (32)	58 (24)	88 (42)	10 g [67]
**n-6:n-3 ratio (<5)** *bDe*	11 (5)	4 (2)	33 (14)	25 (12)	5–10 [66]
**n-6:n-3 ratio (>10)** *BCDE*	76 (35)	67 (33)	21 (9)	6 (3)	
**Fibre** *aCEF*	2 (1)	16 (8)	5 (2)	69 (33)	20 g [66]
**Fibre(USA)** *ACEF*	9 (4)	39 (19)	23 (10)	94 (45)	25 g (f)/38 g (m) [68]
**K** *ABCEF*	9 (4)	37 (18)	40 (17)	81 (39)	3500 mg [66]
**Ca** e	72 (33)	69 (34)	77 (32)	90 (43)	1000 (1200—older 60) mg [66]
**Mg** *AC*d*EF*	13 (6)	63 (31)	33 (14)	92 (44)	420 mg [66]
**P**	11 (5)	16 (8)	7 (3)	8 (4)	700 mg [66]
**Fe** a*CEF*	7 (3)	27 (13)	14 (6)	56 (27)	18 mg (f) 10 mg (m) [66]
**I** b*D*f	94 (43)	100 (49)	72 (30)	92 (44)	150 µg [66]
**Co** bc	4 (2)	16 (8)	26 (11)	23 (11)	10 µg [66]
**Mn**	0 (0)	6 (3)	0 (0)	6 (3)	2 mg [66]
**Cu** *CF*	4 (2)	16 (8)	5 (2)	33 (16)	1 mg [66]
**Mo** ab*C*	78 (36)	96 (47)	98 (41)	100 (48)	70 µg [66]
**Se** a*BCD*e	94 (43)	74 (36)	30 (13)	15 (7)	55 (f)/70 (m) µg [66]
**Cr** *bCE*	72 (33)	84 (41)	95 (40)	100 (48)	40 µg [66]
**Zn**	89 (41)	94 (46)	74 (31)	85 (41)	12 mg [66]
**B_1_** *ACDEF*	13 (6)	59 (29)	12 (5)	88 (42)	1.5 mg [66]
**B_2_** cf	57 (26)	69 (34)	58 (24)	79 (38)	1.8 mg [66]
**PP (B_3_, niacin)** a*cDF*	61 (28)	82 (40)	49 (21)	85 (41)	20 mg [66]
**B_5_** *ABC*d*F*	15 (7)	74 (36)	54 (23)	90 (43)	5 mg [66]
**B_6_** *ABC*e*F*	17 (8)	67 (33)	54 (23)	88 (42)	2 mg [66]
**H (B_7_, folate)**	91 (42)	100 (49)	95 (40)	100 (48)	50 µg [66]
**B_9_ (folate)** *AC*d*EF*	28 (13)	63 (31)	42 (18)	92 (44)	400 µg [66]
**B_12_** b*C*d*E*	100 (46)	100 (49)	84 (35)	69 (33)	3 µg [66]
**C** a*bC*	0 (0)	14 (7)	19 (8)	27 (13)	100 mg [66]
**A (RE)** *ABC*	26 (12)	65 (32)	72 (30)	65 (31)	800 (f)/900 (m) µg RE [66]
**D**	100 (46)	100 (49)	100 (42)	100 (48)	15 (20—older 65) µg [66]
**TE** ab*CEF*	13 (6)	31 (15)	30 (13)	69 (33)	15 mg [66]

A—*p* < 0.001 between VN and LOV, a—*p* < 0.05; B—*p* < 0.001 between VN and FS, b—*p* < 0.05; C—*p* < 0.001 between VN and OMN, c—*p* < 0.05; D—*p* < 0.001 between LOV and FS, d—*p* < 0.05; E—*p* < 0.001 between LOV and OMN, e—*p* < 0.05; F—*p* < 0.001 between FS and OMN, f—*p* < 0.05; *italics*—*p* < 0.05 after Holm-Bonferroni correction.

**Table 3:** Daily dietary nutrient intakes in OMNs and plant-based diets (VNs, LOVs, and FSs, combined), Me (25th percentile; 75th percentile), unadjusted model.

	TOTAL		MALE		FEMALE		Reference Values/Units
Intake/Day	Plant-Based Diets	Omnivorous Diet	Plant-Based Diets	Omnivorous Diet	Plant-Based Diets	Omnivorous Diet	
**n**	137	48	43	16	94	32	
**Age**	34 (30; 37)	34 (29; 41)	33 (31; 39)	33 (29; 37)	35 (29; 49)	35.0 (30.0; 43)	Years
**BMI**	21.3 (20.1; 23.4)	23.7 (22.1; 25.2) **	22.6 (21.0; 24.5)	23.3 (23.0; 24.7)	21.1 (19.6; 23.2)	23.4 (21.2; 25.5) *	kg/m^2^
**Energy value**	2152 (1719; 2849)	1773 (1537; 1946) *	2120 (1716; 2822)	1828 (1768; 2275)	2182 (1720; 2871)	1742 (1370; 1888) *	kcal
**Protein**	57 (48; 79)	69 (59; 74) *	56 (47; 76)	69 (66; 82) *	59 (49; 81)	68 (54; 74)	g
**Fat**	71 (52; 97)	81 (66; 92)	68 (49; 99)	82 (79; 101)	74 (53; 97)	81 (62; 91)	g
**SFA**	15.4 (8.9; 26.1)	29.2 (23.4; 32.6) **	12.8 (8.2; 26.3)	29.2 (25.8; 33.4) **	17.4 (9.0; 26.7)	29.1 (22.9; 32.4) **	g
**MUFA**	13.5 (8.2; 22.6)	12.5 (11.5; 15.1)	14.3 (9.4; 20.4)	13.2 (11.9; 18.4)	13.1 (8.0; 23.2)	12.1 (10.8; 13.2)	g
**PUFA**	21.3 (12.8; 30.7)	16.5 (12.3; 25.1) *	19.3 (11.4; 30.8)	16.8 (15.8; 25.2)	23.5 (13.1; 30.3)	16.4 (10.6; 23.8) *	g
**n-3**	0.7 (0.5; 1.2)	1.1 (0.9; 1.5) **	0.6 (0.4; 1.5)	1.1 (1.0; 2.1) *	0.7 (0.5; 1.2)	1.1 (0.8; 1.3) *	1–3 g [67]
**n-6**	9.0 (5.4; 16.1)	6.5 (5.8; 7.7) *	10.4 (6.1; 14.9)	6.7 (6.3; 9.5)	8.9 (5.1; 16.3)	6.4 (5.5; 7.1) *	10 g [67]
**n-6:n-3 ratio**	11.9 (6.6; 18.9)	6.0 (4.8; 7.4) **	14.1 (6.5; 21.7)	6.2 (4.5; 7.8) **	10.9 (6.7; 17.2)	6.0 (4.8; 7.3) **	5–10 [66]
**Cholesterol**	46 (5; 178)	403 (363; 504) **	23 (3; 125)	408 (322; 565) **	60 (8; 187)	401 (363; 495) **	<300 mg [66]
**Carbohydrate**	328 (241; 450)	182 (114; 233) **	328 (244; 501)	191 (181; 246) *	329 (243; 442)	174 (106; 204) **	g
**MDS**	145 (103; 192)	65 (50; 97) **	150 (84; 236)	66 (62; 109) *	139 (105; 184)	63 (41; 95) **	<75 g [67]
**Fibre**	41 (30; 61)	18 (15; 20) **	43 (30; 63) ^1^	18 (16; 21) ** ^2^	41 (30; 60)	17 (13; 19) **	RF—20 g [66] USA—25 g (f)/38 g (m) [68]
**K**	4774 (3371; 6991)	2634 (2310; 3243) **	4992 (3324; 7297)	2691 (2424; 3429) **	4669 (3467; 6372)	2622 (2270; 3215) **	3500 mg [66]
**Ca**	773 (574; 1058)	736 (638; 842)	723 (556; 969)	740 (701; 859)	789 (581; 1086)	736 (559; 816)	1000 (1200 –older 60) mg [66]
**Mg**	479 (390; 683)	287 (263; 327) **	515 (385; 752)	289 (271; 348) **	477 (393; 668)	287 (230; 304) **	420 mg [66]
**P**	1128 (915; 1504)	1097 (959; 1234)	1086 (877; 1344)	1151 (1026; 1282)	1156 (950; 1544)	1035 (935; 1220)	700 mg [66]
**Fe**	24.5 (18.3; 32.1)	14.6 (13.9; 16.6) **	25.9 (18.4; 39.0)	14.8 (14.3; 18.8) **	23.1 (19.5; 32.3)	14.5 (12.1; 15.3) **	18 mg (f) 10 mg (m) [66]
**I**	61 (39; 93)	72 (65; 87) *	50 (27; 83)	72 (66; 103) *	66 (44; 99)	72 (62; 87)	150 µg [66]
**Co**	23.4 (14.7; 33.1)	12.7 (10.8; 16.9) **	21.2 (12.5; 31.7)	12.1 (11.0; 16.1) *	23.2 (14.6; 36.1)	12.5 (9.9; 17.6) **	10 µg [66]
**Mn**	5.6 (3.9; 7.9)	4.5 (3.6; 6.1) *	5.4 (3.7; 7.1) ^3^	4.9 (4.5; 7.1) ^4^	5.7 (4.0; 8.1)	4.1 (3.3; 5.1) *	2 mg [66]
**Cu**	2.0 (1.4; 2.5)	1.1 (0.9; 1.3) **	1.9 (1.4; 2.4) ^5^	1.1 (1.0; 1.3) ** ^6^	2.0 (1.4; 2.6)	1.0 (0.9; 1.3) **	1 mg [66]
**Mo**	26 (15; 41)	19 (13; 21) **	26 (15; 38)	20 (17; 21) *	26 (15; 42)	19 (12; 22) *	70 µg [66]
**Se**	44 (25; 64)	78 (65; 90) **	37 (23; 63)	82 (78; 111) **	48 (25; 67)	76 (60; 88) **	55 (f)/70 (m) µg [66]
**Cr**	20.6 (2.1; 31.1)	13.6 (10.4; 15.7) **	20.6 (14.1; 31.8)	13.7 (12.7; 15.9) *	20.7 (11.4; 32.0)	13.5 (9.0; 15.2) *	40 µg [66]
**Zn**	6.9 (5.3; 9.4)	7.6 (6.7; 8.4)	6.7 (5.0; 8.3)	7.6 (7.1; 9.4)	7.4 (5.4; 9.5)	7.6 (6.4; 8.4)	12 mg [66]
**B_1_**	1.9 (1.4; 2.5)	1.0 (0.9; 1.2) **	2.0 (1.5; 2.5)	1.1 (1.0; 1.4) **	1.9 (1.4; 2.5)	0.9 (0.9; 1.1) **	1.5 mg [66]
**B_2_**	1.6 (1.2; 2.1)	1.4 (1.3; 1.6) *	1.6 (1.2; 2.2)	1.5 (1.4; 1.8)	1.7 (1.3; 2.0)	1.4 (1.2; 1.6) *	1.8 mg [66]
**PP (B_3_, niacin)**	17.1 (12.4; 22.7)	14.5 (13.0; 16.8) *	17.4 (12.5; 23.2)	15.6 (14.2; 20.0)	17.1 (12.4; 22.8)	14.3 (11.3; 15.1) *	20 mg [66]
**B_5_**	5.1 (3.7; 7.2)	3.8 (3.1; 4.3) **	5.1 (3.9; 7.1)	4.0 (3.2; 4.7) *	5.0 (3.6; 7.3)	3.7 (3.0; 4.2) **	5 mg [66]
**B_6_**	2.0 (1.5; 3.4)	1.4 (1.2; 1.6) **	2.2 (1.5; 3.6)	1.4 (1.3; 1.9) *	2.0 (1.5; 2.8)	1.4 (1.1; 1.6) **	2 mg [66]
**H (B_7_, biotin)**	10.2 (5.7; 18.5)	3.5 (2.9; 4.2) **	10.2 (5.6; 16.0)	3.2 (2.6; 4.4) **	10.3 (5.7; 19.6)	3.5 (3.1; 4.2) **	50 µg [66]
**B_9_ (folate)**	422 (308; 539)	252 (196; 285) **	435 (295; 573)	260 (244; 349) *	422 (313; 530)	241 (169; 266) **	400 µg [66]
**B_12_**	0.03 (0.0; 0.5)	2.3 (1.9; 3.4) **	0.01(0.0; 0.2)	2.3 (2.0; 3.4) **	0.1 (0.0; 0.6)	2.5 (1.8; 3.5) **	3 µg [66]
**C**	223 (147; 423)	127 (82; 153) **	270 (150; 453)	126 (118; 148) **	212 (145; 418)	128 (77; 161) **	100 mg [66]
**A (RE)**	770 (476; 1159)	722 (558; 871)	808 (467; 1200)	725 (517; 921)	767 (481; 1149)	722 (581; 871)	800 (f)/900 (m) µg RE [66]
**D**	0.0 (0.0; 0.3)	0.9 (0.6; 1.1) **	0.0 (0.0; 0.2)	0.9 (0.6; 1.4) **	0.03 (0.0; 0.3)	0.9 (0.6; 1.1) **	15 (20—older 65) µg [66]
**TE**	24.4 (14.8; 32.4)	12.1 (9.3; 17.3) **	27.3 (14.2; 34.9) ^7^	12.9 (10.2; 17.3) ** ^8^	23.1 (15.9; 32.4)	11.8 (8.9; 17.4) **	15 mg TE [66]

Comparisons of values accompanied by a numeric index were made via Student’s t test, since they had a normal distribution. The means ± SDs are as follows: 1—48.13 ± 23.36; 2—18.44 ± 5.17; 3—5.80 ± 2.53; 4—5.99 ± 2.56; 5—2.03 ± 0.95; 6—1.13 ± 0.19; 7—26.73 ± 16.12; and 8—14.25 ± 8.13. * *p* < 0.05; ** *p* < 0.001.

**Table 4:** Daily dietary nutrient intakes in different nutritional groups standardized to 1948 kcal/day, Me (25th percentile; 75th percentile), unadjusted model.

Intake/Day *Significance*	VN	LOV	FS	OMN	Reference Values/Units
**Protein** *CEF*	51 (39; 60)	55 (48; 60)	53 (47; 62)	76 (68; 81)	g
**Fat** *ACD*e*F*	57 (44; 70)	86 (65; 90)	55 (46; 66)	89 (78; 95)	g
**SFA** *ABCDEF*	8.3 (6.7; 10.5)	23.4 (16.2; 28.4)	12.1 (9.77; 18.3)	31.0 (28.5; 35.2)	g
**MUFA** bf	14.7 (10.2; 20.3)	13.8 (9.1; 18.1)	10.4 (6.7; 16.6)	14.2 (12.6; 16.3)	g
**PUFA** *aDe*	17.6 (13.3; 25.4)	25.5 (17.8; 29.1)	14.7 (9.8; 21.1)	18.0 (14.9; 21.8)	g
**n-3** *BCDE*	0.5 (0.4; 0.9)	0.7 (0.5; 0.9)	1.1 (0.6; 1.8)	1.2 (1.1; 1.5)	1–3 g [67]
**n-6** *ce*	9.1 (6.8; 12.5)	9.4 (5.3; 12.0)	7.4 (5.3; 12.7)	7.1 (6.6; 7.9)	10 g [67]
**Cholesterol** *ABCDEF*	3 (1; 8)	189 (77; 270)	50 (25; 88)	448 (410; 538)	<300 mg [66]
**Carbohydrate** *ACDeF*	315 (295; 358)	261 (241; 298)	316 (301; 351)	200 (175; 218)	g
**MDS** *ABCEF*	151 (129; 228)	128 (97; 142)	121 (90; 138)	72 (64; 93)	<75 g [67]
**Fibre** *ABCEF*	53 (46; 62)	32 (27; 39)	32 (27; 37)	19 (17; 21)	RF—20 g [66] USA—25 g (f)/38 g (m) [68]
**K** *ABC*d*E*	6149 (5364; 7416)	3965 (3140; 4708)	3060 (2507; 4214)	2849 (2545; 3182)	3500 mg [66]
**Ca** *aBDeF*	749 (615; 856)	905 (742; 1006)	548 (443; 615)	783 (672; 863)	1000 (1200 –older 60) mg [66]
**Mg** *ABCEF*	600 (540; 695)	427 (367; 516)	383 (331; 469)	307 (283; 345)	420 mg [66]
**P** *aCdF*	999 (866; 1194)	1140 (974; 1214)	992 (331; 469)	1177 (1058; 1352)	700 mg [66]
**Fe** *ABCEf*	28 (23; 33)	21 (17; 24)	18.4 (15.4; 22.6)	16.1 (14.1; 18.3)	18 mg (f) 10 mg (m) [66]
**I** a*CEf*	64 (48; 75)	55 (39; 63)	59 (33; 104)	87 (74; 111)	150 µg [66]
**Co** *aBCde*	26.7 (19.4; 34.1)	19.4 (10.3; 26.7)	11.4 (7.2; 21.7)	13.0 (10.2; 15.9)	10 µg [66]
**Mn** *BcDf*	5.7 (5.1; 7.0)	5.4 (4.3; 6.2)	4.1 (3.3; 5.6)	5.0 (4.5; 5.3)	2 mg [66]
**Cu** *ABCEf*	2.2 (1.9; 2.6)	1.6 (1.2; 2.0)	1.4 (1.1; 2.0)	1.1 (1.0; 1.3)	1 mg [66]
**Mo** *aBCDE*f	34.7 (25.2; 64.5)	28.1 (18.7; 38.4)	11.1 (5.7; 20.1)	19.1 (13.3; 22.4)	70 µg [66]
**Se** *ABCdEF*	23 (12; 34)	43 (35; 51)	63 (42; 77)	87 (73; 96)	55 (f)/70 (m) µg [66]
**Cr** *aBCDE*f	28.4 (19.2; 44.8)	22.5 (15.1; 29.9)	9.4 (4.4; 13.3)	13.9 (10.0; 15.7)	40 µg [66]
**Zn** *acEf 6*	6.7 (5.9; 8.7)	6.0 (5.1; 7.0)	6.5 (4.7; 8.4)	8.5 (7.4; 9.8)	12 mg [66]
**B_1_** *A*b*CdEF*	2.0 (1.7; 2.4)	1.5 (1.2; 1.9)	1.8 (1.4; 2.1)	1.1 (1.0; 1.2)	1.5 mg [66]
**B_2_** *BDF*	1.5 (1.3; 1.8)	1.6 (1.4; 1.8)	1.3 (1.1; 1.5)	1.5 (1.4; 1.6)	1.8 mg [66]
**PP (B_3_, niacin)** *Ad*e	16.3 (14.7; 19.7)	14.3 (11.9; 16.3)	16.1 (13.5; 18.8)	16.0 (14.3; 17.7)	20 mg [66]
**B_5_** *ABC*	6.3 (5.2; 7.3)	4.0 (3.6; 5.1)	4.2 (3.1; 5.2)	4.1 (3.4; 4.8)	5 mg [66]
**B_6_** *ABCd*	3.0 (2.3; 3.8)	1.7 (1.2; 2.1)	1.5 (1.1; 2.0)	1.5 (1.2; 1.7)	2 mg [66]
**H (B_7_, biotin)** *ABCEF*	14.1 (9.6; 21.3)	7.5 (4.3; 14.6)	7.5 (4.3; 12.6)	3.7 (2.8; 5.1)	50 µg [66]
**B_9_ (folate)** *a*b*CEf*	444 (365; 548)	356 (315; 434)	361 (258; 445)	274 (236; 301)	400 µg [66]
**B_12_** *ABCEF*	0.0 (0.0; 0.01)	0.3 (0.1; 0.5)	0.1 (0.0; 1.3)	2.6 (2.1; 3.8)	3 µg [66]
**C** *ABC*d*E*	377 (330; 476)	219 (145; 285)	123 (86; 182)	136 (98; 155)	100 mg [66]
**A (RE)** *BcDF*	975 (767; 1301)	817 (545; 1167)	367 (222; 654)	816 (637; 1029)	800 (f)/900 (m) µg RE [66]
**D** *ABCDEF*	0.0 (0.0; 0.0)	0.3 (0.1; 0.7)	0.0 (0.0; 0.2)	1.0 (0.7; 1.3)	15 (20—older 65) µg [66]
TE * *BCDEF*	24.6 (20.3; 29.8)	24.1 (19.6; 27.8)	15.4 (13.1; 22.4)	13.4 (10.9; 16.6)	15 mg TE [66]

* TE intake values were normally distributed in all the groups, the means ± SD were as follows: VN—25.12 ± 8.27; LOV—23.86 ± 8.40; FS—17.65 ± 7.36; OMN—14.12 ± 5.37. Comparison of peach-colored cells was performed via Student’s t test. A—*p* < 0.001 between VN and LOV, a—*p* < 0.05; B—*p* < 0.001 between VN and FS, b—*p* < 0.05; C—*p* < 0.001 between VN and OMN, c—*p* < 0.05; D—*p* < 0.001 between LOV and FS, d—*p* < 0.05; E—*p* < 0.001 between LOV and OMN, e—*p* < 0.05; F—*p* < 0.001 between FS and OMN, f—*p* < 0.05; *italics*—*p* < 0.05 after Holm-Bonferroni correction.

## Data Availability

The data presented in this study are available on request from the corresponding author (the data are not publicly available due to privacy).

## Supplementary Materials

The following supporting information can be downloaded at: https://www.mdpi.com/article/10.3390/foods14061062/s1, Supplementary Table S1. Anthropometric parameters of the subjects; Supplementary Table S2. Two-way analysis of variance of the influence of diet and age on nutrient intakes; Supplementary Table S3, Three-way analysis of variance of the influence of diet, gender, and BMI on nutrient intakes; Supplementary Table S4, Correlation analysis of associations between BMI and calorie adjusted nutrient intakes, found in three-factor rank analysis of variation (Supplementary Table S3), unadjusted model; Supplementary Table S5. Daily dietary nutrient intakes in different nutritional groups in the lower quartile, unadjusted model; Supplementary Table S6. Daily dietary nutrient intakes in different nutritional groups in the upper quartile, unadjusted model; Supplementary Table S7. Correlation analysis between the plant-based diet adherence (VN, LOV, or FS) and absolute or calorie adjusted nutrient intakes, unadjusted model; Supplementary Table S8. Four-way analysis of variance of the influence of diet (plant-based or omnivorous), gender, age, and BMI on nutrient intakes.

## Appendix A

Finally: we need to emphasise again that the purpose of this study was to compare the diets themselves: plant-based ones (VN, LOV, and FS) with OMN diet. Our study aimed not to compare the subjects’ health or their micronutrient status (vitamin or elemental content of any biosubstrates). Consequently, since the subjects’ diets were the objects of the study, we did not analyse their lifestyle, physical activity level, bad habits, intake of supplements or pharmacological preparations, comorbidities (except for acute diseases, such subjects were not included), education level, religious beliefs, marriage status, average monthly income, or other medical or socioeconomic parameters. Obviously, vegetarianism is not only a group of diets, it is a whole philosophy that fundamentally defines a lifestyle. The same, of course, is true of a Christian Lent. A person who observes religious fasting is likely to be deeply religious, which significantly distinguishes him from the average OMN secular person of a modern metropolis in various aspects of life. On the one hand, this is a serious limitation of our study, since ignoring all of the above factors deprived us of the opportunity to speculate about differences in health, well-being, or mentality between these four groups of subjects, which have been observed and described in the literature. Alewaeters et al. [177] have reported a lower incidence of smoking, less frequent alcohol consumption, and greater physical activity among Flemish vegetarians in both genders. However, no differences were observed in physical activity or smoking in the U.S. study [178]. No differences were found in physical activity level between LOV and OMN women in Taiwan [179]. Vegans from the EPIC-Oxford cohort were the main supplement users among men. Women from all the groups (VN, LOV, pescetarian, and OMN) took supplements more often than men, even VNs, but no differences were found between them [57]. At the same time, Schüpbach et al. [74], even could not recruit enough participants not taking supplements to fulfil the VN group in their study, unlike LOV and OMN groups. Thus, we may assume that in our study supplement use might have more impacted the micronutrient status in VNs than in LOVs or OMNs. Regarding sociodemographic characteristics, Alles et al. [86] reported a higher education level in LOVs compared to VNs and OMNs (though VNs were significantly younger than other groups) and the highest average income in OMNs, but this group was the least presented in the 18–30 years age interval. Therefore, such data do not look too reliable.

Much less data are available on FSs in this regard. According to Sarri et al. [41], FSs are much less likely smokers than non-FS subjects, whereas the authors did not reveal differences in education level. The same patterns were supported in 2023 [82]. The authors did not observe differences in supplement use, physical activity, night sleeping, or reading, as well. However, they found less time spent on watching TV or using computer or mobile phone in FSs.

On the other hand, focusing only on diet gave us the opportunity to fully explore diet as a separate and independent factor influencing health. Not including dietary supplements in the calculations allowed us to assess the contribution of diet alone to nutrient intake, which demonstrates the reliability of such diets as a source of nutrients and will provide practitioners with a clear understanding of the strengths and weaknesses of each diet in order to make dietary recommendations to such patients. At the same time, hypothetically, those taking supplements could limit their nutritional intake and vice versa, those not taking them could consume more food to avoid nutritional deficiencies. However, this assumption needs corresponding investigations not to be speculative.
