# Supplementary material for: Nutrient Intakes in Vegans, Lacto-Ovo-Vegetarians, Orthodox Fasters, and Omnivores in Russia: A Cross-Sectional Study
Source: Foods. 2025 Mar 20;14(6):1062. doi: 10.3390/foods14061062 (PMC11942464; doi:10.3390/foods14061062)
Supplement: Supplementary file 1 [file foods-14-01062-s001.zip › Supplementary Table 8.pdf]

Supplementary Table 8. Four-way analysis of variance of the influence of diet (plant-based or omnivorous), gender, age ,and BMI on nutrient intakes, p-value.

| **Nutrient intake/** ***calorie adjusted nutrient intake*** | **Diet** | **Gender** | **Age** | **BMI** | **Diet + Gender** | **Diet + BMI** | **Age + Gender** | **Gender + BMI** | **Age + BMI** | **Diet + Gender + Age** | **Diet + Gender + BMI** | **Diet + Age + BMI** | **Gender + Age + BMI** | **Diet + Gender + Age + BMI** | **Full regression** | **MRSC** |
|---|---|---|---|---|---|---|---|---|---|---|---|---|---|---|---|---|
| **EV** | 0.395 | 0.197 | 0.437 | 0.596 | 0.083 | 0.453 | 0.35 | 0.378 | 0.222 | 0.109 | 0.132 | 0.193 | 0.229 | 0.105 | 0186 | **<0.001** |
| **Protein** | 0.114 | 0.242 | 0.894 | 0.471 | 0.074 | 0.332 | 0.2 | 0.159 | 0.052 | **0.028** | **0.037** | 0.243 | 0.221 | 0.1221 | 0.086 | **<0.001** |
| | *0.74* | *0.424* | *0.704* | *0.533* | *0.208* | *0.357* | *0.327* | *0.391* | *0.072* | *0.095* | *0.136* | *0.127* | *0.354* | *0.26* | *0.443* | ***<0.001*** |
| **Fat** | 0.687 | 0.392 | 0.834 | 0.164 | 0.22 | 0.667 | 0.305 | 0.764 | 0.062 | 0.242 | 0.177 | 0.621 | 0.321 | 0.084 | 0.067 | **<0.001** |
| | *0.817* | *0.39* | *0.751* | *0.406* | *0.25* | *0.713* | *0.301* | *0.71* | *0.055* | *0.275* | *0.202* | *0.148* | *0.394* | *0.145* | *0.44* | ***<0.001*** |
| **SFA** | **0.001** | 0.257 | 0.369 | 0.557 | 0.104 | 0.963 | 0.141 | 0.443 | **0.011** | **0.026** | 0.145 | 0.219 | **0.049** | **0.024** | <0.001 | **<0.001** |
| | *0.056* | *0.222* | *0.458* | *0.702* | *0.142* | *0.768* | *0.214* | *0.441* | ***0.01*** | *0.09* | *0.148* | ***0.032*** | *0.155* | *0.082* | *0.117* | ***<0.001*** |
| **MUFA** | 0.358 | 0.631 | 0.239 | 0.465 | 0.444 | 0.709 | 0.866 | 0.963 | 0.406 | 0.656 | 0.36 | 0.637 | 0.817 | 0.421 | 0.362 | **<0.001** |
| | *0.524* | *0.498* | *0.806* | *0.642* | *0.412* | *0.893* | *0.677* | *0.974* | *0.202* | *0.46* | *0.254* | *0.495* | *0.78* | *0.341* | *0.643* | ***<0.001*** |
| **PUFA** | 0.55 | 0.487 | 0.408 | 0.289 | 0.511 | 0.673 | 0.242 | 0.764 | 0.552 | 0.656 | 0.316 | 0.873 | 0.545 | 0.082 | 0.087 | **<0.001** |
| | *0.185* | *0.45* | *0.465* | *0.403* | *0.472* | *0.652* | *0.34* | *0.812* | *0.477* | *0.68* | *0.29* | *0.469* | *0.616* | *0.233* | *0.282* | ***<0.001*** |
| **n3** | 0.057 | 0.419 | 0.726 | 0.532 | 0.262 | 0.718 | 0.337 | 0.947 | 0.295 | 0.326 | 0.675 | 0.291 | 0.167 | **0.012** | 0.06 | **<0.001** |
| | *0.428* | *0.325* | *0.321* | *0.462* | *0.310* | *0.522* | *0.283* | *0.842* | *0.205* | *0.281* | *0.225* | *0.122* | *0.472* | *0.055* | *0.394* | ***<0.001*** |
| **n6** | **0.003** | 0.893 | 0.093 | 0.21 | 0.894 | 0.696 | 0.548 | 0.426 | 0.737 | 0.823 | 0.498 | 0.128 | 0.398 | 0.958 | **0.007** | **<0.001** |
| | ***0.034*** | *0.854* | *0.487* | *0.395* | *0.924* | *0.852* | *0.809* | *0.504* | *0.494* | *0.754* | *0.349* | *0.602* | *0.798* | *0.748* | *0.1* | ***<0.001*** |
| **n-6:n-3 ratio** | **<0.001** | 0.915 | 0.13 | 0.977 | 0.45 | 0.628 | 0.668 | 0.68 | 0.75 | 0.414 | 0.976 | 0.329 | 0.479 | 0.641 | **0.003** | **<0.001** |
| **Cholesterol** | **<0.001** | 0.06 | 0.251 | 0.314 | **0.012** | 0.107 | 0.071 | **0.021** | **0.005** | **0.006** | **0.002** | 0.133 | **0.029** | **0.008** | **<0.001** | **<0.001** |
| | ***<0.001*** | *0.132* | *0.303* | *0.696* | ***0.042*** | *0.383* | *0.056* | *0.0609* | ***0.003*** | ***0.022*** | ***0.017*** | ***0.013*** | *0.132* | ***0.029*** | ***<0.001*** | ***<0.001*** |
| **Carbohydrate** | **0.008** | 0.307 | 0.351 | 0.993 | 0.16 | 0.247 | 0.609 | 0.459 | 0.634 | 0.219 | 0.3 | 0.127 | 0.274 | 0.237 | **<0.001** | **<0.001** |
| | *0.209* | *0.595* | *0.493* | *0.77* | *0.317* | *0.339* | *0.688* | *0.636* | *0.49* | *0.248* | *0.411* | *0.189* | *0.395* | *0.473* | *0.212* | ***<0.001*** |
| **MDS** | **0.022** | 0.371 | 0.498 | 0.774 | 0.438 | 0.431 | 0.72 | 0.638 | 0.73 | 0.416 | 0.416 | 0.114 | 0.38 | 0.197 | **0.015** | **<0.001** |
| | *0.241* | *0.551* | *0.585* | *0.931* | *0.49* | *0.503* | *0.78* | *0.804* | *0.61* | *0.37* | *0.431* | *0.171* | *0.438* | *0.467* | *0.559* | ***<0.001*** |
| **Fibre** | **<0.001** | 0.951 | 0.756 | 0.582 | 0.888 | 0.947 | 0.922 | 0.922 | 0.697 | 0.841 | 0.766 | 0.722 | 0.897 | 0.85 | **<0.001** | **<0.001** |
| | ***0.033*** | *0.901* | *0.91* | *0.683* | *0.736* | *0.667* | *0.999* | *0.927* | *0.977* | *0.558* | *0.603* | *0.604* | *0.689* | *0.908* | *0.22* | ***<0.001*** |

| **Nutrient intake/** *calorie adjusted nutrient intake* | Diet | Gender | Age | BMI | Diet + Gender | Diet + BMI | Age + Gender | Gender + BMI | Age + BMI | Diet + Gender + Age | Diet + Gender + BMI | Diet + Age + BMI | Gender + Age + BMI | Diet + Gender + Age + BMI | Full regression | MRSC |
|---|---|---|---|---|---|---|---|---|---|---|---|---|---|---|---|---|
| **K** | **0.001** | 0.887 | 0.636 | 0.696 | 0.803 | 0.989 | 0.897 | 0.783 | 0.818 | 0.94 | 0.692 | 0.642 | 0.99 | 0.729 | **0.009** | **<0.001** |
| | *0.08* | *0.832* | *0.982* | *0.699* | *0.663* | *0.72* | *0.865* | *0.881* | *0.814* | *0.574* | *0.559* | *0.507* | *0.738* | *0.753* | *0.552* | ***<0.001*** |
| **Ca** | 0.204 | 0.627 | 0.602 | 0.071 | 0.516 | 0.786 | 0.975 | 0.848 | 0.193 | 0.313 | 0.287 | 0.819 | 0.208 | 0.476 | 0.253 | **<0.001** |
| | *0.515* | *0.565* | *0.91* | *0.403* | *0.425* | *0.762* | *0.764* | *0.881* | *0.165* | *0.3* | *0.309* | *0.426* | *0.334* | *0.515* | *0.66* | ***<0.001*** |
| **Mg** | **<0.001** | 0.849 | 0.444 | 0.488 | 0.803 | 0.958 | 0.973 | 0.796 | 0.969 | 0.879 | 0.621 | 0.815 | 0.992 | 0.641 | **<0.001** | **<0.001** |
| | ***0.037*** | *0.795* | *0.854* | *0.54* | *0.673* | *0.65* | *0.882* | *0.923* | *0.654* | *0.525* | *0.488* | *0.545* | *0.752* | *0.696* | *0.261* | ***<0.001*** |
| **P** | 0.761 | 0.432 | 0.975 | 0.309 | 0.118 | 0.474 | 0.338 | 0.312 | 0.09 | 0.091 | 0.073 | 0.454 | 0.276 | **0.033** | 0.134 | **<0.001** |
| | *0.685* | *0.523* | *0.8* | *0.469* | *0.26* | *0.44* | *0.45* | *0.566* | *0.107* | *0.166* | *0.18* | *0.227* | *0.407* | *0.296* | *0.44* | ***<0.001*** |
| **Fe** | **0.003** | 0.583 | 0.651 | 0.894 | 0.476 | 0.686 | 0.494 | 0.459 | 0.728 | 0.743 | 0.533 | 0.419 | 0.513 | 0.528 | **0.005** | **<0.001** |
| | *0.117* | *0.69* | *0.623* | *0.781* | *0.461* | *0.526* | *0.638* | *0.696* | *0.595* | *0.432* | *0.456* | *0.346* | *0.482* | *0.632* | *0.375* | ***<0.001*** |
| **I** | 0.852 | 0.667 | 0.604 | 0.541 | 0.254 | 0.621 | 0.232 | 0.312 | 0.376 | 0.535 | 0.836 | 0.78 | 0.98 | 0.635 | 0.253 | **<0.001** |
| | *0.976* | *0.927* | *0.65* | *0.576* | *0.227* | *0.535* | *0.186* | *0.312* | *0.192* | *0.266* | *0.45* | *0.538* | *0.836* | *0.53* | *0.486* | ***<0.001*** |
| **Co** | **0.008** | 0.497 | 0.852 | 0.767 | 0.713 | 0.992 | 0.689 | 0.976 | 0.892 | 0.952 | 0.829 | 0.954 | 0.842 | 0.803 | **0.047** | **<0.001** |
| | *0.084* | *0.95* | *0.87* | *0.702* | *0.486* | *0.634* | *1* | *0.868* | *0.643* | *0.495* | *0.587* | *0.654* | *0.551* | *0.932* | *0.3* | ***<0.001*** |
| **Mn** | 0.503 | 0.513 | 0.724 | 0.98 | 0.226 | 0.966 | 0.565 | 0.805 | 0.771 | 0.585 | 0.438 | 0.804 | 0.532 | 0.958 | 0.807 | **<0.001** |
| | *0.6* | *0.691* | *0.842* | *0.821* | *0.355* | *0.86* | *0.514* | *0.822* | *0.513* | *0.391* | *0.381* | *0.468* | *0.49* | *0.816* | *0.914* | ***<0.001*** |
| **Cu** | **0.001** | 0.942 | 0.832 | 0.695 | 0.461 | 0.665 | 0.61 | 0.796 | 0.751 | 0.603 | 0.348 | 0.605 | 0.79 | 0.598 | **<0.001** | **<0.001** |
| | *0.082* | *0.871* | *0.737* | *0.703* | *0.512* | *0.543* | *0.682* | *0.881* | *0.626* | *0.439* | *0.384* | *0.497* | *0.68* | *0.683* | *0.295* | ***<0.001*** |
| **Mo** | 0.055 | 0.811 | 0.594 | 0.948 | 0.82 | 0.815 | 0.931 | 0.959 | 0.906 | 0.717 | 0.777 | 0.574 | 0.804 | 0.845 | 0.452 | **<0.001** |
| | *0.292* | *0.963* | *0.626* | *0.961* | *0.599* | *0.933* | *0.802* | *0.959* | *0.881* | *0.81* | *0.62* | *0.516* | *0.656* | *0.789* | *0.918* | ***0.004*** |
| **Se** | **<0.001** | **0.043** | **0.038** | 0.664 | **0.015** | 0.134 | 0.077 | 0.094 | 0.081 | **0.023** | **0.021** | **0.037** | 0.154 | **0.047** | **0.002** | **<0.001** |
| | *0.128* | *0.21* | *0.121* | *0.965* | *0.1* | *0.215* | *0.149* | *0.281* | *0.121* | *0.082* | *0.082* | ***0.043*** | *0.321* | *0.122* | *0.452* | ***<0.001*** |
| **Cr** | **0.036** | 0.972 | 0.688 | 0.909 | 0.831 | 0.915 | 0.926 | 0.929 | 0.967 | 0.733 | 0.701 | 0.509 | 0.905 | 0.691 | 0.334 | **<0.001** |
| | *0.228* | *0.844* | *0.604* | *0.965* | *0.59* | *0.842* | *0.786* | *0.888* | *0.864* | *0.738* | *0.555* | *0.448* | *0.676* | *0.666* | *0.87* | ***<0.001*** |
| **Zn** | 0.085 | 0.121 | 0.316 | 0.86 | **0.034** | 0.091 | 0.067 | 0.056 | 0.85 | **0.02** | **0.012** | **0.028** | 0.16 | 0.057 | 0.276 | **<0.001** |
| | *0.797* | *0.331* | *0.455* | *0.813* | *0.153* | *0.223* | *0.262* | *0.34* | *0.126* | *0.088* | *0.921* | *0.068* | *0.269* | *0.208* | *0.507* | ***<0.001*** |
| **$B_1$** | **<0.001** | 0.51 | 0.858 | 0.537 | 0.376 | 0.407 | 0.642 | 0.622 | 0.917 | 0.464 | 0.424 | 0.573 | 0.568 | 0.527 | **<0.001** | **<0.001** |
| | *0.063* | *0.655* | *0.658* | *0.055* | *0.464* | *0.331* | *0.706* | *0.785* | *0.68* | *0.362* | *0.417* | *0.422* | *0.531* | *0.625* | *0.093* | ***<0.001*** |

| **Nutrient intake/** ***calorie adjusted nutrient intake*** | Diet | Gender | Age | BMI | Diet + Gender | Diet + BMI | Age + Gender | Gender + BMI | Age + BMI | Diet + Gender + Age | Diet + Gender + BMI | Diet + Age + BMI | Gender + Age + BMI | Diet + Gender + Age + BMI | Full regression | MRSC |
|---|---|---|---|---|---|---|---|---|---|---|---|---|---|---|---|---|
| **$B_2$** | 0.43 | 0.268 | 0.993 | 0.73 | 0.127 | 0.709 | 0.474 | 0.29 | 0.171 | 0.201 | 0.077 | 0.547 | 0.398 | 0.215 | 0.407 | **<0.001** |
| | *0.849* | *0.485* | *0.791* | *0.658* | *0.271* | *0.55* | *0.512* | *0.568* | *0.169* | *0.221* | *0.096* | *0.252* | *0.465* | *0.354* | *0.658* | ***<0.001*** |
| **PP ($B_3$, niacin)** | 0.891 | 0.143 | 0.458 | 0.913 | **0.025** | 0.166 | 0.26 | 0.077 | 0.48 | 0.121 | **0.047** | 0.083 | 0.438 | 0.196 | 0.176 | **<0.001** |
| | *0.662* | *0.489* | *0.508* | *0.805* | *0.19* | *0.304* | *0.442* | *0.375* | *0.346* | *0.175* | *0.205* | *0.135* | *0.453* | *0.388* | *0.49* | ***<0.001*** |
| **$B_5$** | **0.016** | 0.845 | 0.371 | 0.55 | 0.514 | 0.882 | 0.815 | 0.681 | 0.668 | 0.647 | 0.545 | 0.875 | 0.974 | 0.927 | 0.089 | **<0.001** |
| | *0.127* | *0.816* | *0.872* | *0.57* | *0.526* | *0.6* | *0.769* | *0.773* | *0.724* | *0.372* | *0.442* | *0.526* | *0.701* | *0.824* | *0.477* | ***<0.001*** |
| **$B_6$** | **0.005** | 0.755 | 0.326 | 0.756 | 0.992 | 0.65 | 0.873 | 0.63 | 0.496 | 0.924 | 0.983 | 0.689 | 0.704 | 0.661 | **0.027** | **<0.001** |
| | *0.069* | *0.762* | *0.76* | *0.702* | *0.786* | *0.89* | *0.819* | *0.776* | *0.986* | *0.604* | *0.663* | *0.473* | *0.958* | *0.674* | *0.566* | ***<0.001*** |
| **H ($B_7$, biotin)** | **<0.001** | 0.44 | 0.203 | 0.351 | 0.676 | 0.834 | 0.521 | 0.466 | 0.523 | 0.907 | 0.801 | 0.497 | 0.958 | 0.391 | **0.003** | **<0.001** |
| | ***0.012*** | *0.709* | *0.473* | *0.416* | *0.957* | *0.781* | *0.608* | *0.613* | *0.83* | *0.714* | *0.935* | *0.731* | *0.71* | *0.52* | *0.109* | ***0.002*** |
| **$B_9$** | **0.007** | 0.384 | 0.738 | 0.907 | 0.174 | 0.51 | 0.669 | 0.423 | 0.807 | 0.47 | 0.346 | 0.56 | 0.659 | 0.556 | **0.002** | **<0.001** |
| | *0.141* | *0.714* | *0.722* | *0.857* | *0.374* | *0.57* | *0.746* | *0.764* | *0.835* | *0.432* | *0.451* | *0.485* | *0.624* | *0.689* | *0.452* | ***<0.001*** |
| **$B_{12}$** | **<0.001** | 0.566 | 0.382 | 0.821 | 0.13 | 0.577 | 0.075 | 0.11 | 0.097 | 0.071 | 0.062 | 0.306 | 0.808 | 0.116 | **<0.001** | **<0.001** |
| | ***<0.001*** | *0.305* | *0.376* | *0.867* | *0.071* | *0.519* | *0.087* | *0.194* | *0.042* | *0.156* | *0.104* | *0.108* | *0.528* | ***0.033*** | ***<0.001*** | ***<0.001*** |
| **C** | **0.001** | 0.854 | 0.298 | 0.54 | 0.754 | 0.838 | 0.613 | 0.703 | 0.441 | 0.631 | 0.853 | 0.946 | 0.687 | 0.804 | **0.025** | **<0.001** |
| | *0.052* | *0.984* | *0.587* | *0.634* | *0.986* | *0.956* | *0.793* | *0.701* | *0.748* | *0.968* | *0.71* | *0.96* | *0.96* | *0.854* | *0.656* | ***0.002*** |
| **A (RE)** | 0.387 | 0.967 | 0.92 | 0.557 | 0.484 | 0.52 | 0.868 | 0.565 | 0.486 | 0.599 | 0.506 | 0.973 | 0.824 | 0.717 | 0.795 | **<0.001** |
| | *0.633* | *0.912* | *0.76* | *0.765* | *0.432* | *0.591* | *0.796* | *0.62* | *0.421* | *0.368* | *0.496* | *0.546* | *0.618* | *0.731* | *0.927* | ***0.001*** |
| **D** | **<0.001** | 0.112 | 0.421 | 0.346 | **0.033** | 0.506 | 0.239 | 0.082 | 0.05 | **0.021** | **0.016** | 0.554 | **0.038** | **0.025** | **<0.001** | **<0.001** |
| | ***<0.001*** | *0.133* | *0.349* | *0.557* | ***0.035*** | *0.732* | *0.132* | *0.113* | ***0.008*** | ***0.031*** | ***0.036*** | *0.063* | *0.104* | ***0.032*** | ***<0.001*** | ***<0.001*** |
| **E (TE)** | **<0.001** | 0.992 | 0.99 | 0.238 | 0.928 | 0.773 | 0.695 | 0.555 | 0.736 | 0.916 | 0.559 | 0.718 | 0.896 | 0.357 | **<0.001** | **<0.001** |
| | ***0.023*** | *0.786* | *0.832* | *0.388* | *0.667* | *0.665* | *0.631* | *0.746* | *0.594* | *0.827* | *0.455* | *0.691* | *0.833* | *0.407* | *0.117* | ***<0.001*** |

EV – Enegry value;

MRSC – multiple regression shift coefficient.
