# Supplementary material for: Nutrient Intakes in Vegans, Lacto-Ovo-Vegetarians, Orthodox Fasters, and Omnivores in Russia: A Cross-Sectional Study
Source: Foods. 2025 Mar 20;14(6):1062. doi: 10.3390/foods14061062 (PMC11942464; doi:10.3390/foods14061062)
Supplement: Supplementary file 1 [file foods-14-01062-s001.zip › Supplementary Tables 1-7.pdf]

Supplementary Table 1. Anthropometric parameters of the subjects, Me (25th percentile; 75th percentile) or n (%).

Intake/day	VN	LOV	FS	OMN	Reference values / Units
**n (♀ + ♂)** *ab*	46 (24 + 22)	49 (37 + 12)	42 (33 + 9)	48 (32 + 16)	-
**% (♀ + ♂)**	52 + 48	76 + 24	79 + 21	67 + 33	
**Age**	31 (29; 33)	35 (30; 54)	41 (34; 49)	34 (29; 41)	Years
**Hight** *abc*	1.75 (1.68; 1.79)	1.66 (1.63; 1.73)	1.68 (1.62; 1.75)	1.66 (1.63; 1.75)	m
**Weight** *de*	64.5 (57.8; 71.3)	59.2 (52.0; 69.3)	64.5 (57.7; 75.4)	66.9 (62.3; 74.7)	kg
**BMI** *BCdE*	21.0 (19.8; 22.7)	21.3 (19.5; 23.5)	23.5 (21.2; 25.4)	23.7 (22.9; 25.0)	18.5 – 25 kg/m^2
**Underweight, n (%)**	5 (11)	4 (8)	1 (2)	3 (6)	< 18.5 kg/m^2
**Overweight, n (%)** *Bc*	1 (2)	6 (12)	12 (29)	8 (17)	25 – 29.9 kg/m^2
**Obese, n (%)**	1 (2)	2 (4)	2 (5)	3 (6)	> 30 kg/m^2
**Overweight + Obese, n (%)** *Bc*	2 (4)	8 (16)	14 (33)	11 (23)	> 25 kg/m^2
**BMI out of normal range, n (%)** b	7 (15)	12 (24)	15 (36)	14 (29)	< 18.5 kg/m^2 or > 25 kg/m^2

VN – vegans; LOV – lacto-ovo-vegetarians; FS – fasters; OMN – omnivores; BMI – body mass index.
The values accompanied by * had a normal distribution; the means ± SDs were as follows: Height (VN: 1.73 ± 0.08; FS: 1.69 ± 0.09); BMI (FS: 23.8 ± 3.7).
Comparison of peach-coloured cells was performed via Student's t test.
A - p<0.001 between VN and LOV, a - p<0.05;
B - p<0.001 between VN and FS, b - p<0.05;
C - p<0.001 between VN and OMN, c - p<0.05;
D - p<0.001 between LOV and FS, d - p<0.05;
E - p<0.001 between LOV and OMN, e - p<0.05;
F - p<0.001 between FS and OMN, f - p<0.05;
italics - p<0.05 after Holm-Bonferroni correction.

Supplementary Table 2. Two-way analysis of variance of the influence of diet and age on nutrient intakes, p-value. 13

**Nutrient intake/ *calorie adjusted nutrient intake***	**Diet**	**Age**	**Diet+Age**
**E**	0.058	0.261	0.474
**Pretein**	**0.011**	0.874	0.39
	0.322	*0.828*	*0.545*
**Fat**	0.209	0.291	0.603
	0.779	*0.731*	*0.782*
**SFA**	**<0.001**	0.466	0.519
	0.049	*0.911*	*0.711*
**MUFA**	0.081	0.978	0.586
	0.1	*0.941*	*0.654*
**PUFA**	**0.025**	0.093	0.307
	0.074	*0.361*	*0.78*
**n3**	**<0.001**	0.523	0.851
	0.008	*0.847*	*0.749*
**n6**	**0.002**	0.869	0.568
	0.005	*0.995*	*0.583*
**n-6:n-3 ratio**	**<0.001**	0.525	0.884
**Cholesterol**	**<0.001**	0.559	0.478
	<0.001	*0.88*	*0.635*
**Carbohydrate**	**<0.001**	0.255	0.98
	0.001	*0.27*	*0.172*
**MDS**	**<0.001**	0.29	0.167
	0.001	*0.286*	*0.125*
**Fibre**	**<0.001**	0.502	0.26
	<0.001	*0.356*	*0.24*
**K**	**<0.001**	0.43	0.134
	<0.001	*0.293*	*0.117*
**Ca**	0.223	0.136	0.446
	0.213	*0.386*	*0.524*
**Mg**	**<0.001**	0.835	0.322
	<0.001	*0.573*	*0.465*
**P**	0.542	0.348	0.388
	0.253	*0.494*	*0.514*
**Fe**	**<0.001**	0.461	0.265
	<0.001	*0.458*	*0.29*
**I**	**<0.001**	0.437	0.666
	0.054	*0.775*	*0.809*
**Co**	**<0.001**	0.244	0.307
	<0.001	*0.145*	*0.099*
**Mn**	0.061	**0.036**	0.352
	0.15	*0.313*	*0.696*
**Cu**	**<0.001**	0.375	0.255
	<0.001	*0.302*	*0.236*
**Mo**	**<0.001**	0.05	0.094
	0.002	*0.14*	*0.131*
**Se**	**<0.001**	0.068	0.07
	0.003	*0.389*	*0.287*

**Nutrient intake/**<br>***calorie adjusted nutrient intake***	Diet	Age	Diet+Age
**Cr**	**<0.001**	0.055	0.084
	<0.001	*0.092*	*0.065*
**Zn**	**0.004**	0.851	0.097
	0.083	*0.664*	*0.255*
**B_1**	**<0.001**	0.642	0.583
	<0.001	*0.382*	*0.382*
**B_2**	**0.04**	0.413	0.353
	0.072	*0.449*	*0.453*
**PP (B_3, niacin)**	**<0.001**	0.298	0.121
	0.02	*0.316*	*0.264*
**B_5**	**<0.001**	0.801	0.378
	<0.001	*0.536*	*0.256*
**B_6**	**<0.001**	0.96	0.337
	<0.001	*0.489*	*0.185*
**H (B_7, biotin)**	**<0.001**	0.293	0.69
	<0.001	*0.967*	*0.486*
**B_9 (folate)**	**<0.001**	0.227	0.336
	0.001	*0.364*	*0.56*
**B_{12}**	**<0.001**	0.784	0.94
	<0.001	*0.955*	*0.878*
**C**	**<0.001**	0.587	0.28
	<0.001	*0.513*	*0.417*
**A (RE)**	**0.003**	**0.027**	**0.008**
	0.019	***0.042***	***0.004***
**D**	**<0.001**	0.229	0.623
	<0.001	*0.559*	*0.63*
**E (TE)**	**<0.001**	0.089	0.651
	<0.001	*0.298*	*0.799*

Supplementary Table 3. Three-way analysis of variance of the influence of diet, gender, and BMI on nutrient intakes, p-value.

**Nutrient intake/ *calorie adjusted nutrient intake***	**Diet**	**Gender**	**BMI**	**Diet + Gender**	**Diet + BMI**	**Gender + BMI**	**Diet + Gender + BMI**
**Energy value**	**<0.001**	0.086	0.313	0.506	0.059	0.257	0.613
**Protein**	**<0.001**	0.782	**0.002**	0.666	**0.024**	0.246	0.422
	0.008	*0.830*	*0.113*	*0.829*	*0.058*	*0.148*	*0.501*
**Fat**	0.348	0.824	**0.043**	0.692	**0.039**	**0.032**	0.241
	0.260	*0.745*	*0.184*	*0.695*	*0.165*	***0.034***	*0.298*
**SFA**	**<0.001**	0.954	**0.001**	0.649	**0.013**	**0.023**	0.171
	0.058	*0.648*	*0.051*	*0.619*	*0.101*	*0.061*	*0.375*
**MUFA**	0.23	0.593	0.587	0.614	0.068	0.151	0.64
	0.124	*0.669*	*0.896*	*0.643*	*0.138*	*0.057*	*0.429*
**PUFA**	0.109	0.827	0.767	0.743	0.147	**0.021**	0.24
	0.042	*0.714*	*0.868*	*0.534*	*0.357*	***0.01***	*0.178*
**n-3**	**<0.001**	0.401	**0.007**	0.471	0.066	0.062	0.133
	<0.001	*0.416*	***0.015***	*0.328*	*0.064*	***0.038***	*0.069*
**n-6**	**0.003**	0.701	0.194	0.936	**0.006**	0.055	0.316
	0.003	*0.772*	*0.426*	*0.987*	***0.016***	***0.02***	*0.171*
**n-6:n-3ratio**	**<0.001**	0.179	**<0.001**	0.426	**<0.001**	0.627	0.226
**Cholesterol**	**<0.001**	0.581	**<0.001**	0.743	**<0.001**	0.084	**0.043**
	<0.001	*0.932*	***0.002***	*0.797*	***0.035***	*0.259*	*0.415*
**Carbohydrate**	**<0.001**	0.967	0.531	0.538	**0.033**	0.738	0.901
	<0.001	*0.682*	*0.973*	*0.738*	***0.04***	*0.352*	*0.641*
**MDS**	**<0.001**	0.515	0.225	0.634	**0.022**	0.883	0.739
	<0.001	*0.931*	*0.881*	*0.752*	*0.07*	*0.494*	*0.617*
**Fibre**	**<0.001**	0.975	**0.028**	0.42	**<0.001**	0.817	0.836
	<0.001	*0.686*	*0.51*	*0.626*	***0.016***	*0.423*	*0.802*
**K**	**<0.001**	0.943	0.108	0.359	**<0.001**	0.499	0.45
	<0.001	*0.821*	*0.848*	*0.598*	***0.02***	*0.755*	*0.655*
**Ca**	0.198	0.869	0.118	0.776	**0.01**	0.2	0.482
	0.271	*0.966*	*0.294*	*0.685*	*0.140*	*0.108*	*0.546*
**Mg**	**<0.001**	0.795	0.156	0.638	**0.004**	0.732	0.843
	<0.001	*0.947*	*0.797*	*0.832*	***0.049***	*0.441*	*0.820*
**P**	**0.049**	0.539	**0.028**	0.736	**0.015**	0.241	0.539
	0.018	*0.761*	*0.214*	*0.81*	***0.036***	*0.128*	*0.519*
**Fe**	**<0.001**	0.833	0.297	0.475	**0.006**	0.713	0.828
	<0.001	*0.633*	*0.927*	*0.638*	*0.052*	*0.517*	*0.8296*
**I**	**<0.001**	**0.034**	0.016	0.497	0.317	0.499	0.976
	<0.001	*0.116*	*0.055*	*0.726*	*0.456*	*0.784*	*0.94*
**Co**	**<0.001**	**0.048**	0.137	0.148	**0.022**	0.809	0.177
	<0.001	*0.216*	*0.894*	*0.246*	***0.016***	*0.626*	*0.389*
**Mn**	0.076	0.502	0.642	0.228	0.180	0.222	0.312
	0.2	*0.472*	*0.834*	*0.495*	*0.323*	*0.161*	*0.587*
**Cu**	**<0.001**	0.265	0.221	0.315	**0.002**	0.494	0.718
	<0.001	*0.428*	*0.852*	*0.561*	***0.028***	*0.316*	*0.787*
**Mo**	**<0.001**	0.267	0.061	0.189	**0.008**	0.629	0.268
	**<0.001**	*0.391*	*0.518*	*0.231*	*0.118*	*0.602*	*0.512*

**Nutrient intake/** ***calorie adjusted nutrient intake***	Diet	Gender	BMI	Diet + Gender	Diet + BMI	Gender + BMI	Diet + Gender + BMI
**Se**	**<0.001**	0.760	**<0.001**	**0.044**	**<0.001**	0.063	**0.023**
	<0.001	*0.961*	*0.081*	*0.502*	***0.041***	*0.069*	*0.219*
**Cr**	**<0.001**	0.577	0.052	0.35	**0.001**	0.834	0.238
	<0.001	*0.579*	*0.599*	*0.359*	***0.028***	*0.733*	*0.381*
**Zn**	**<0.001**	0.718	**0.011**	0.656	**0.038**	0.559	0.43
	0.004	*0.749*	*0.158*	*0.719*	*0.058*	*0.217*	*0.78*
**B_1**	**<0.001**	0.966	0.794	0.670	0.075	0.892	0.943
	<0.001	*0.753*	*0.802*	*0.837*	***0.019***	*0.286*	*0.768*
**B_2**	**0.048**	0.961	0.667	0.714	**0.022**	0.392	0.546
	0.023	*0.85*	*0.625*	*0.79*	***0.046***	*0.18*	*0.583*
**PP (B_3, niacin)**	**<0.001**	0.798	0.297	0.578	**0.024**	0.9	0.541
	0.001	*0.593*	*0.563*	*0.750*	***0.049***	*0.401*	*0.671*
**B_5**	**<0.001**	0.735	0.657	0.216	**0.046**	0.662	0.739
	<0.001	*0.642*	*0.823*	*0.586*	***0.039***	*0.588*	*0.8*
**B_6**	**<0.001**	0.187	0.096	0.372	**0.008**	**0.039**	0.23
	<0.001	*0.671*	*0.764*	*0.697*	*0.051*	*0.653*	*0.486*
**H (B_7, biotin)**	**<0.001**	0.296	0.193	0.124	0.059	0.668	0.326
	<0.001	*0.394*	*0.689*	*0.291*	***0.039***	*0.377*	*0.789*
**B_9 (folate)**	**<0.001**	0.827	0.218	0.433	**0.019**	0.763	0.825
	<0.001	*0.555*	*0.608*	*0.602*	***0.1***	*0.444*	*0.858*
**B_{12}**	**<0.001**	0.325	**<0.001**	0.86	**<0.001**	0.413	0.516
	<0.001	*0.95*	***<0.001***	*0.735*	***0.01***	*0.294*	*0.632*
**C**	**<0.001**	0.795	0.065	0.345	**<0.001**	0.840	0.834
	**<0.001**	*0.923*	*0.526*	*0.53*	*0.052*	*0.513*	*0.947*
**A (RE)**	**0.001**	0.290	0.137	0.339	**<0.001**	0.766	**0.045**
	0.025	*0.342*	*0.248*	*0.357*	***0.001***	*0.872*	*0.082*
**D**	**<0.001**	0.664	**<0.001**	0.812	**<0.001**	0.216	0.251
	<0.001	*0.935*	***0.0127***	*0.634*	***0.020***	*0.286*	*0.573*
**TE**	**<0.001**	0.899	0.115	0.916	**0.042**	0.066	0.308
	<0.001	*0.979*	*0.605*	*0.799*	*0.205*	***0.04***	*0.3*

SFA – saturated fatty acids; MUFA – mono-unsaturated fatty acids; PUFA – poly-unsaturated fatty acids; MDS – mono- and disaccharides; RE – retinol equivalents; TE – tocopherol equivalents.

Non-Italics – absolute terms, *Italics* – calorie adjusted terms.

Supplementary Table 4. Correlation analysis of associations between BMI and calorie adjusted nutrient intakes, found in three-factor rank analysis of variation (Supplementary Table S3), unadjusted model.

	**n-6:n-3 ratio**		**n-3 adj**		**Cholesterol adj**		**Vitamin B_{12} adj**		**Vitamin D adj**	
	r	p-value	r	p-value	r	p-value	r	p-value	r	p-value
**BMI**	-0.18	0.008	0.127	0.043	0.146	0.25	0.167	0.012	0.114	0.062

r – Spearman correlation coefficient;
adj – calorie adjusted nutrient intake.

Supplementary Table 5. Daily dietary nutrient intakes in different nutritional groups in the lower quartile, unadjusted model, Me.

**Intake/day** *Significance*	**VN**	**LOV**	**FS**	**OMN**	**Reference values/Units**
**Energy value** *AbCd*f	1405	1116	1500	1247	kcal
**Protein** b*cDe*	32	27	48	52	g
**Fat** c	31	41	36	54	g
**SFA** *A*b*C*d*EF*	3.7	9.7	7.0	18.9	g
**MUFA** ab*EF*	7.0	4.6	5.0	10.3	g
**PUFA**	8.9	10.3	7.1	7.7	g
**n-3** *BCdE*	0.3	0.4	0.7	0.7	1-3 g [67]
**n-6** a*e*	5.4	2.7	3.8	5.1	10 g [67]
**n-6:n-3ratio** bc*DE*	5.0	6.6	3.2	2.9	5 - 10 [66]
**Cholesterol** *ABCD*e*F*	0.4	38.7	16.3	42.2	<300 mg [66]
**Carbohydrate** *ACDEF*	210	156	256	103	g
**MDS** *aCEF*	86	60	74	38	<75 g [67]
**Fibre** *A*b*CDEF*	37	16.7	24.3	10.8	RF – 20 g [66] USA - 25 g (f) / 38 g (m) [68]
**K** *AbCF*	4000	2326	2794	1972	3500 mg [66]
**Ca** b	518	518	401	414	1000 (1200 –older 60) mg [66]
**Mg** *AbCdf*	385	239	320	210	420 mg [66]
**P** *a*b*d*	714	571	820	806	700 mg [66]
**Fe** bCd*F*	19.7	12.2	15.2	11.1	18 mg (f) 10 mg (m) [66]
**I** *CEF*	30	24	27	59	150 µg [66]
**Co** *ABC*	16.4	7.1	6.2	7.7	10 µg [66]
**Mn** *AcD*f	3.7	2.5	3.5	3.1	2 mg [66]
**Cu** *acdF*	1.5	0.8	1.3	0.8	1 mg [66]
**Mo** *aBcD*ef	20.9	11.7	5.2	8.8	70 µg [66]
**Se** *ABCDE*	8	20	35	52	55 (f) / 70 (m) µg [66]
**Cr** a*BcD*ef	17.6	9.8	4.2	6.6	40 µg [66]
**Zn** abc*DE*	4.9	3.0	5.0	5.7	12 mg [66]
**B_1** *ACDF*	1.3	0.9	1.4	0.8	1.5 mg [66]
**B_2**	1.1	0.9	1.1	1.2	1.8 mg [66]
**PP (B_3, niacin)** *Ab*c*DeF*	11.5	7.9	14.6	10.3	20 mg [66]
**B_5** *ABCDf*	4.8	2.1	3.3	2.7	5 mg [66]
**B_6** *ABC*d	1.9	0.9	1.1	1.0	2 mg [66]
**H (B_7, biotin)** *AbC*f	6.6	2.7	3.2	2.4	50 µg [66]
**B_9 (folate)** *ACD*e*F*	296	215	287	153	400 µg [66]
**B_{12}** *ABCDEF*	0.0	0.02	0.0	1.6	3 µg [66]
**C** *ACef*	180	89	89	58	100 mg [66]
**A (RE)** *bDF*	391	423	210	421	800 (f) / 900 (m) µg RE [66]
**D** *ACDEF*	0.0	0.0	0.0	0.5	15 (20 – older 65) µg [66]
**TE** a*cef*	13.8	9.9	9.8	6.5	15 mg TE [66]

A - p<0.001 between VN and LOV, a - p<0.05;
B - p<0.001 between VN and FS, b - p<0.05;
C - p<0.001 between VN and OMN, c - p<0.05;
D - p<0.001 between LOV and FS, d - p<0.05;
E - p<0.001 between LOV and OMN, e - p<0.05;
F - p<0.001 between FS and OMN, f - p<0.05;
italics - p<0.05 after Holm-Bonferroni correction.

Supplementary Table 6. Daily dietary nutrient intakes in different nutritional groups in the upper quartile, unadjusted model, Me.

**Intake/day** *Significance*	**VN**	**LOV**	**FS**	**OMN**	**Reference values / Units**
**Energy value** a*BD*e*f*	3322	3029	3925	2362	kcal
**Protein** b*D*f	97	82	116	96	g
**Fat** *bf*	109	125	144	111	g
**SFA** *AbC*	21	35	38	38	g
**MUFA** cef	33	29	31	19	g
**PUFA** *Ce*f	41	38	45	28	g
**n-3** *bcDE*	1.3	1.2	2.7	2.5	1-3 g [67]
**n-6** *CEF*	22.6	20.2	22.3	10.1	10 g [67]
**n-6:n-3 ratio** a*BC*d*EF*	40	25	16	9	5 - 10 [66]
**Cholesterol** *ABCEf*	26	330	365	614	<300 mg [66]
**Carbohydrate** *ACDeF*	580	429	609	281	g
**MDS** *A*b*CF*	429	182	286	138	<75 g [67]
**Fibre** *ABC*d*EF*	87	56	69	23	RF – 20 g [66] USA - 25 g (f) / 38 g (m) [68]
**K** *A*b*CEF*	11343	6128	6958	3732	3500 mg [66]
**Ca** *CeF*	1298	1356	1524	985	1000 (1200 –older 60) mg [66]
**Mg** *A*b*CEF*	1147	708	794	378	420 mg [66]
**P** *D*f	1729	1617	2080	1417	700 mg [66]
**Fe** *A*b*C*d*EF*	48	29	37	19	18 mg (f) 10 mg (m) [66]
**I** *AbDEF*	132	81	280	117	150 µg [66]
**Co** ab*CEF*	54	40	39	19.7	10 µg [66]
**Mn** *d*e	11.1	11.7	8.3	8.5	2 mg [66]
**Cu** a*CEF*	3.9	2.4	3.2	1.3	1 mg [66]
**Mo** *aBC*d*EF*	88	58	34	23	70 µg [66]
**Se** *aBCDef*	50	70	145	111	55 (f) / 70 (m) µg [66]
**Cr** *aBCdEf*	69	41	26	18	40 µg [66]
**Zn** a*D*	11.9	9.2	15.8	12.4	12 mg [66]
**B_1** *ACDEF*	3.9	2.5	3.8	1.6	1.5 mg [66]
**B_2** a*c*de*f*	2.8	2.3	2.8	1.9	1.8 mg [66]
**PP (B_3, niacin)** a*c*d*F*	26	23	31	20	20 mg [66]
**B_5** *ACd*e*F*	11.1	6.5	10.5	4.8	5 mg [66]
**B_6** *ABCDeF*	6.2	2.6	3.6	2.0	2 mg [66]
**H (B_7, biotin)** *A*b*CEF*	44.8	20.2	23.2	4.7	50 µg [66]
**B_9 (folate)** a*C*d*EF*	704	548	712	358	400 µg [66]
**B_{12}** *ABCDE*	0.0	0.7	3.7	4.1	3 µg [66]
**C** *aBCEF*	774	407	366	183	100 mg [66]
**A (RE)** *ABC*	2234	1481	1200	1215	800 (f) / 900 (m) µg RE [66]
**D** *AbCdEF*	0.0	0.9	0.3	1.5	15 (20 – older 65) µg [66]
**TE** *CEF*	45	41	37	22	15 mg TE [66]

A - p<0.001 between VN and LOV, a - p<0.05;
B - p<0.001 between VN and FS, b - p<0.05;
C - p<0.001 between VN and OMN, c - p<0.05;
D - p<0.001 between LOV and FS, d - p<0.05;
E - p<0.001 between LOV and OMN, e - p<0.05;
F - p<0.001 between FS and OMN, f - p<0.05;
italics - p<0.05 after Holm-Bonferroni correction.

Supplementary Table 7. Correlation analysis between the plant-based diet adherence (VN, LOV, or FS) and absolute or calorie adjusted nutrient intakes, unadjusted model.

	r	p-value	r	p-value	r	p-value	r	p-value
	**Energy value**				**n-6:n-3 ratio**			
**Abs**	0.178	0.008			0.344	0.000		
	**Protein**		**Fat**		**SFA**		**MUFA**	
**Abs**	-0.104	0.078	-0.076	0.152	-0.335	0.000	0.091	0.108
**Adj**	0.037	0.310	0.009	0.452	-0.149	0.021	0.105	0.077
	**PUFA**		**n-3 PUFA**		**n-6 PUFA**		**Cholesterol**	
**Abs**	0.184	0.006	-0.219	0.001	0.250	0.000	-0.655	0.000
**Adj**	0.165	0.012	-0.057	0.22	0.217	0.001	-0.364	0.000
	**Carbohydrate**		**MDS**		**Fibre**		**K**	
**Abs**	0.394	0.000	0.344	0.000	0.475	0.000	0.375	0.000
**Adj**	0.224	0.001	0.206	0.002	0.278	0.000	0.238	0.001
	**Ca**		**Mg**		**P**		**Fe**	
**Abs**	0.093	0.104	0.417	0.000	0.076	0.152	0.381	0.000
**Adj**	0.094	0.102	0.266	.000	0.103	0.081	0.239	0.001
	**I**		**Co**		**Mn**		**Cu**	
**Abs**	0.003	0.485	0.318	0.000	0.147	0.023	0.406	0.000
**Adj**	0.057	0.218	0.241	0.000	0.122	0.049	0.252	0.000
	**Mo**		**Se**		**Cr**		**Zn**	
**Abs**	0.243	0.000	-0.289	0.000	0.269	0.000	-0.034	0.322
**Adj**	0.169	0.011	-0.051	0.245	0.191	0.005	0.081	0.136
	**B_1**		**B_2**		**PP (B_3, niacin)**		**B_5**	
**Abs**	0.429	0.000	0.153	0.019	0.178	0.008	0.305	0.000
**Adj**	0.263	0.000	0.134	0.034	0.153	0.019	0.220	0.001
	**B_6**		**H (B_7, biotin)**		**B_9 (folate)**		**B_{12}**	
**Abs**	0.313	0.000	0.372	0.000	0.391	0.000	-0.580	0.000
**Adj**	0.237	0.001	0.274	0.000	0.238	0.001	-0.335	0.000
	**C**		**A (RE)**		**D**		**TE**	
**Abs**	0.345	0.000	0.118	0.054	-0.609	0.000	0.383	0.000
**Adj**	0.226	0.001	0.106	0.076	-0.376	0.000	0.261	0.000

r – Spearman correlation coefficient;
abs – absolute nutrient intake;
adj – calorie adjusted nutrient intake.
